# The MOBSTER R package for tumour subclonal deconvolution from bulk DNA whole-genome sequencing data

**DOI:** 10.1186/s12859-020-03863-1

**Published:** 2020-11-17

**Authors:** Giulio Caravagna, Guido Sanguinetti, Trevor A. Graham, Andrea Sottoriva

**Affiliations:** 1 University of Trieste, Trieste, Italy; 2 International School for Advanced Studies, Trieste, Italy; 3 Barts Cancer Institute, Barts and the London School of Medicine and Dentistry, Queen Mary University of London, London, UK; 4 The Institute of Cancer Research, London, UK

**Keywords:** Tumour subclonal deconvolution, Cancer evolution, Population genetics, Dirichlet mixture model, Whole-genome DNA sequencing

## Abstract

**Background:**

The large-scale availability of whole-genome sequencing profiles from bulk DNA sequencing of cancer tissues is fueling the application of evolutionary theory to cancer. From a bulk biopsy, subclonal deconvolution methods are used to determine the composition of cancer subpopulations in the biopsy sample, a fundamental step to determine clonal expansions and their evolutionary trajectories.

**Results:**

In a recent work we have developed a new model-based approach to carry out subclonal deconvolution from the site frequency spectrum of somatic mutations. This new method integrates, for the first time, an explicit model for neutral evolutionary forces that participate in clonal expansions; in that work we have also shown that our method improves largely over competing data-driven methods. In this Software paper we present mobster, an open source R package built around our new deconvolution approach, which provides several functions to plot data and fit models, assess their confidence and compute further evolutionary analyses that relate to subclonal deconvolution.

**Conclusions:**

We present the mobster package for tumour subclonal deconvolution from bulk sequencing, the first approach to integrate Machine Learning and Population Genetics which can explicitly model co-existing neutral and positive selection in cancer. We showcase the analysis of two datasets, one simulated and one from a breast cancer patient, and overview all package functionalities.

## Background

One of the most exciting recent developments in cancer informatics is the ability to reconstruct the evolutionary history and clonal composition of tumours from whole-genome DNA sequencing (WGS) data [1, 2]. This analysis leverages statistical models and bioinformatics tools that can recapitulate patient-level *intra-tumour heterogeneity*, and that we can use to study, from an evolutionary point of view, tumour evolutionary patterns across multiple patients [3–6]. An investigation of the evolutionary forces underpinning a tumour usually begins by performing a *subclonal deconvolution* of the bulk WGS data of a single patient. The main objective of this first and crucial task is to determine how many cancer (sub)clones co-exist in the tumour, and the general architecture of tumour population [7]. This step is crucial as the construction of the tumour evolutionary trajectory depends on results from this analysis. The deconvolution of the signal is however challenged by the mixed effect of positive, neutral and negative selection that are all potentially operating within the tumour; see a review and references therein for a discussion on the role of these forces in driving tumour evolution [8].

Several unsupervised bioinformatics tools exist that can be used to determine the clonal architecture of a tumour; all of the tools solve different formulations of a clustering problem, defined from the site frequency spectrum of somatic mutations detected in the WGS biopsies and other covariates [7]. In a recent work [9], we have shown that these clustering methods have severe limitations stemming from the fact that they do not account for neutral evolutionary forces, one key force underpinning tumour growth (see [10–13]). A direct consequence of their data-driven design is that they tend to overestimate the number of tumour clones, and the complexity of the tumour clonal architecture. In the same work [9], we have mitigated these limitations by approaching the clustering problem through the integration of Machine Learning and Population Genetics. In particular, we have used mathematical models from Population Genetics to create a mixture model that, for the first time, could account also for within-clone neutral evolutionary dynamics.

**Fig. 1:**
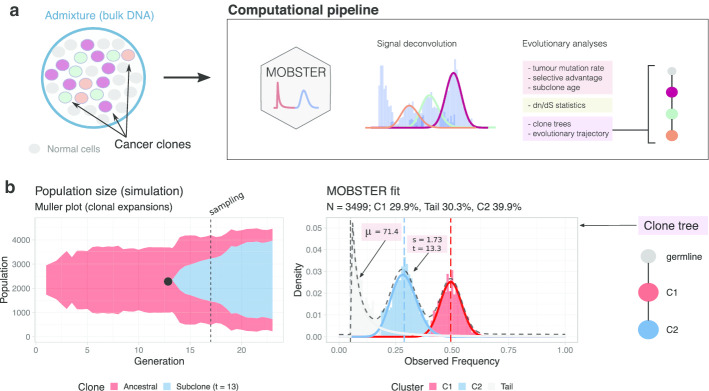
**a** Pipeline for data analysis with the mobster R package for subclonal deconvolution. The package can be used to infer the clonal composition of a tumour bulk DNA biopsy. The statistical method integrates machine learning and evolutionary theory to detect subclones that have expanded due to positive selection, while modelling intra-clone neutral evolution with distributions predicted by population genetics. mobster also computes clone-specific evolutionary parameters (e.g., mutation rates, selection coefficients), dN/dS statistics and clone trees. **b** Example tumour clonal expansion simulated with the stochastic branching process implemented in the TEMULATOR (https://t-heide.github.io/TEMULATOR/) package. After $t=13$ cell doublings a subclonal driver triggers a clonal expansion that sweeps in the ancestral population (left). Using mobster from a bulk sample collected at $t=17$ we can retrieve the tumour architecture, the clone tree and the evolutionary parameters of the simulated tumour (right)

The new model-based method MOBSTER (Fig. 1a) can identify subclones that are experiencing *positive selection,* while at the same time modelling background neutral dynamics. This Software paper describes the implementation of mobster, a new open source R package for tumour subclonal deconvolution that implements the statistical model introduced in [9].

## Implementation

The package mobster can be used to detect cancer subclones in bulk whole-genome DNA sequencing assays of tumour and matched normal samples (Fig. 1a). Besides clustering, the package implements several other analyses that can compute a number of evolutionary parameters that characterize tumour growth, which we discuss below.

The package is implemented using the S3 object system in R (version >= 3.6.0), providing easy access to the main inference algorithm and a number of visualization functions that can be used to inspect the input data, and the model fits. The package supports different types of input formats that store input data for the somatic mutations detected in the tumour. The theory of MOBSTER is based on the site frequency spectrum of each somatic mutation, which can be either single-nucleotide variants or more complex insertions and deletions (provided the frequency spectrum is computable). For every mutation mobster needs to know either the Variant Allele Frequency (VAF), i.e., the ratio of read counts mapping to the mutant allele, over the total coverage at the variant locus, or the Cancer Cell Fraction (CCF). The CCF is the proportion of cancer cells harboring the mutations, and must be pre-computed normalizing the VAF for tumour sample purity and tumour copy number segments. All the calls (i.e., somatic mutations, copy number and tumour purity estimates) should be generated before tumour subclonal analysis using external bioinformatics tools. Input frequency values have to range in $[\mathrm{0,1}]$ consistently with the support of the probability distributions used in MOBSTER’s statistical model. VAF values by definition range in $[\mathrm{0,1}]$. For CCF values this is not necessarily the case, since CCF values of clonal mutations, which are by definition present in 100% of cancer cells, range around 1; for this reason, canonical CCF values can be adjusted by dividing the CCF estimate by half. In this case they technically represent the expected allele frequency for a clonal diploid mutation, for a pure tumour sample (i.e., a sample with no contamination from normal cells).

To facilitate data input for the mobster package, input VAF values can also be provided from a file storing somatic mutations in the standard Variant Calling Format (see the package manual for input requirements).

### The model

The statistical model implemented in the mobster package a Dirichlet finite mixture with mixed distributions [9]. It contains one (optional) Pareto Type-I random variable (a type of power-law), and $k\ge 1$ Beta random variables; the overall model is a univariate finite mixture with $k+1$ components if the tail is fit to data, and $k$ otherwise. In this model, Beta components capture ongoing clonal expansions, while the power law tail captures neutral dynamics; the power law distribution is predicted by Population Genetics of mutant alleles spreading in growing populations, and has been recently used within cancer evolution [10–12]. Model selection determines the optimal value for $k$, and also if a tail should be fit, or not, to data. A model fit with $k=1$ represents a monoclonal tumour (i.e., with no evidence of ongoing subclonal selection), a model with $k>1$ is polyclonal.

The likelihood for $n$ datapoints ${x}_{i}$ in data $D$ is

$$f(D|\theta ,\pi )={\prod\limits_{i=1}^{n}}\left[{\pi }_{1}g({x}_{i}|{x}_{*},\alpha ) + {\sum\limits_{w=2}^{k}}{\pi }_{w}h({x}_{i}|{a}_{w-1},{b}_{w-1})\right]$$

where $g$ and $h$ are density functions for the mixture components, $\theta =\{{x}_{*},\alpha ,{a}_{1},\ldots,{a}_{k},{b}_{1},\ldots, {b}_{k}\}$ is a set of parameters; here $\pi =({\pi }_{1}, \ldots,{\pi }_{k+1})$ are mixing proportions in a standard mixture model with $n\times (k+1)$ latent variables $z$ (which determine the assignment probability of each one of the $n$ input points to the $k+1$ mixture components). The Pareto component (fixed indexed position $1$ of the mixture) follows the standard Pareto Type-I density function, and the other components the standard Beta density. The details on the densities, the model derivation and the fitting strategy are presented in detail in [9].

A MOBSTER model is fit through an iterative procedure that resembles an Expectation-Maximization strategy; the fit combines the maximum likelihood estimators for the Pareto tail, and the moment-matching estimator for the Beta peaks (default implementation). A full maximum likelihood estimation via the expectation maximization algorithm is also available. Model selection can optimize the value of $k$ *as well as* the presence or absence of the tail in the fit. A model $M$ with size $\lambda$ (number of parameters) is scored according to the following quantities

$$\text{NLL}= -log\, f\left(D|\theta ,\pi \right) \qquad (\text{negative log-likelihood})$$

$$\text{BIC}=2\,NLL+\lambda\,log\,n \qquad (\text{Bayesian Information Criterion})$$

$$\text{ICL}=\text{BIC}+H\left(z\right) \qquad (\text{Integrated Classification Likelihood})$$

$$\text{reICL}=\text{BIC}+H\left(\widehat{z}\right) \qquad (\text{reduced ICL}).$$

Here $H(z)$ is the entropy of the latent variables $z$, and $\widehat{z}$ a re-normalisation of $z$ after removal of tail mutations (i.e., points with hard clustering assignments to the tail). The scoring is obtained extending the popular BIC with the entropy of the model's latent variables, which leads to the ICL approach. We also derived a different version of the ICL score which uses the entropy for a subset of variables $\widehat{z}$; this latter is the default score for model selection and is called reduced ICL (reICL). The intuition of reICL is to penalize the overlap between Beta components of the mixture—i.e., promoting models with clear, well-separated subclonal peaks.

In [9] we provide extensive testing for MOBSTER, comparing our approach to other popular methods in the field in a variety of settings; e.g., with single-sample or multi-region tumor datasets, with variable sequencing coverage and sample purity, and with different input clonal architectures.

### Main software functions

#### Fitting functions

Function mobster_fit is the interface to the Dirichlet finite mixture that can cluster the input mutations into $k$ Beta components—i.e., $k$ clonal peaks—and one optional power law tail for neutral mutations. The function implements a routine for model-selection that determines the optimal number of Beta clusters $k\ge 1$, and whether a tail should be used to fit the data, or not. Models are scored using data likelihood, regularised by model complexity with the aid of the entropy of the latent variables (see above); this function takes a parameter to identify which scoring strategy should be used to determine the best model. Function mobster_fit repeats the fit a desired number of times, sampling multiple initial conditions for the model parameters to avoid local optima. The tool can exploit a parallel inference engine, which can be used to speed up the fit with multi-core architectures. In general, however, the fit is fast compared to other approaches, possibly because of the maximum-likelihood formulation of the inference, e.g., the analysis of a tumour with about 13,000 somatic mutations takes about one minute, on a standard laptop, without exploiting the parallel inference engine.

A post-hoc cluster-selection heuristic is available to filter out clusters that are either too small, or that contain too few mutations; post-hoc, the package can also assign new mutations (i.e., previously unseen) to the model’s clusters, conditioning on the inferred parameters. Density functions are available for the S3 model object, as well as functions to sample data from a fit model, or to create a random generative model (clusters and parameters) that can be used for data generation. This utility can be easily used for synthetic benchmarking of MOBSTER or other subclonal deconvolution tools.

Confidence of the fits can be computed using parametric and non-parametric bootstrap procedures that are available in mobster [14]. Both procedures take as input a model object computed from mobster_fit, and the number $n$ of required bootstrap samples. The non-parametric approach re-samples $n$ datasets of size equal to the original dataset, therefore sampling with repetition from the original dataset; the parametric approach samples $n$ new datasets from the fit model. In both cases the new datasets are used to fit $n$ new models with a parallel fitting routing, and from the output fits the package computes a distribution over the full model (i.e., the frequency at which the input model is re-fit) and over the parameter fits to the original data. Bootstrap results can be used to estimate confidence intervals, using a given confidence $\alpha$-level with $0<\alpha <1$ to determine quantiles. In the case of the non-parametric approach the package also computes the co-clustering probability for each pair of input mutations; this is a quantity that can be used to determine clustering’s stability, defined as the frequency at which two mutations cluster together. We note that in this case the frequency is bounded by the probability of sampling every pair of mutations in the same bootstrap resample.

#### Visualization functions

Model fits can be plotted as data histograms colored by clustering assignment, with the fit density (per component and overall) overlaid to the data histogram. A number of functions can be used to plot the model likelihood, the entropy of the latent variables, the sum of squares error of the fit to data and the mixing proportions. An additional function allows the user to inspect alternative fits of the data, which helps for model selection in cases where one is not confident about the tool parameterizations. Specific functions also allow to visualize results from bootstrap analysis, giving easy access to the bootstrap distribution and confidence intervals.

#### Post-clustering analyses

The package can be used to run further (post-clustering) analyses directly from the output of mobster_fit. For example, dN/dS statistics from the ratio of nonsynonymous to synonymous single nucleotide mutations can be computed for either the clusters of a single patient, or across multiple patients (e.g., pooling all tail mutations). Computations are carried out interfacing mobster to dndscv, an R package for dN/dS [15]. From a fit, mobster can compute also the tumour’s evolutionary parameters, revealing the *mutation rate* $\mu >0$, and the *selective advantage coefficient* $s>0$ and the age of each subclone, in units of tumour doubling times. Interfacing with the ctree R package, clone trees can also be readily assembled from the output mobster clusters [4].

#### Detailed functionalities

In Additional file 1 of this paper we provide six extra notes that explain the most relevant mobster functions and their parameters.

Using a markdown-style with example R code, these notes discuss the following topics:

1. Introduction to the input format, simple fits and data-generation process;
2. plotting functions for fit models and input data;
3. bootstrap estimation for confidence assessment;
4. post-clustering inference of Population Genetics parameters of tumour growth;
5. post-clustering clone-specific dN/dS statistics;
6. post-clustering clone trees generation.

## Results

### Relation to tumour growth models

We show the analysis of a simulated tumour using the mobster package. The tumour is simulated using thee *stochastic branching process* [11–13] model of tumour growth, which is implemented in the TEMULATOR open source R package.^1^

In brief, the stochastic branching process that we use is a discrete-time discrete-state Markov process that describes cell divisions and mutation accumulation. At each time point $t>0$ cells divide or die, with some probability; when they divide successfully the somatic mutations are copied into daughter cells, and new accumulation are accumulated at a certain rate (the tumour mutation rate). This simple linear birth–death model captures the diffusion of mutant alleles (i.e., somatic mutations) in expanding populations (i.e., cells dividing). A selective advantage coefficient $s>0$ controls the propensity of successful cell divisions or, in other words, the rate of growth of the progeny of any given cell—this is a measure of fitness for a cellular population. In the model implemented in the TEMULATOR package, the cell initiating the tumour starts with a baseline value ${s}_{0}$, and its progeny keeps sharing the same ${s}_{0}$ value. At any given timepoint, if a new subclonal driver mutation triggers the formation of a more fit clone, its value of $s$ is increased relative to ${s}_{0}$ (i.e. ${s}_{1}={s}_{0}+{\delta }_{1}$ is the new clone-specific value for the coefficient). It is possible to show that, in the long run, the new subclonal population outgrows the parental clonal population. This model is particularly interesting since it allows deriving the analytical Power law distribution for within-clone neutral evolutionary dynamics used in the MOBSTER model [9]—i.e., the distribution of the site frequency spectrum for somatic mutations that accumulate in-between the formation of new clones with different values of $s$. This is true for both the deterministic version of the model based on an ordinary differential equation representation of the Markov chain [11, 12], as well as for the stochastic counterpart ruled by a master equation [13].

In Fig. 1b (left panel) we show a simulated tumour where after $t=13$ cell doublings a subclonal driver triggers a clonal expansion that sweeps in the ancestral population. The tumour bulk WGS data is simulated form the tumour cell population collected at time $t=17$, when the subclone has reached a tumour mass that is detectable relative to the overall tumour size, considering a simulated WGS assay with Poisson-distributed coverage with rate $\lambda =120$ (i.e., mean coverage 120×).

We run filtering of simulated somatic mutations consistently with a standard quality-check analysis, and identify $n=3499$ somatic mutations with VAF above 5%. We use these to run a mobster analysis with default parameters Fig. 1b (right panel), and retrieve the generative model simulated with TEMULATOR. In particular, the tool detects $K=2$ Beta clusters, plus one Pareto tail. The Beta clusters reveal clonal mutations (cluster C1, red; 30% of the mutations) present in all tumour cells in the simulated biopsy, as well as the mutations that characterize the ongoing subclonal expansion (cluster C2, blue; about 40% of the mutations). Mutations assigned to the tumour tail—an intermixing of the tails of both clones—are about 30% of the simulated mutations. Using functionalities of the mobster package we retrieve evolutionary parameters that we used to simulate the tumour with TEMULATOR. In particular we obtain a mutation rate $\mu =71.4$ (in mutations per cell division), and the subclone parameters—i.e., we date the subclone to $\widehat{t}=13.3$ (age of the subclone relative to its ancestor) and retrieve its selection coefficient to $\widehat{s}=1.73$ (where 1 is the baseline value of the ancestor). Through the interface of mobster with other packages, we can also retrieve the clone tree that explains this architecture, which in this case is trivial because C2 is the only possible descendant of C1.

Overall, this simple example shows that we can simulate tumour growth (with specific evolutionary parameters like mutation rate, selection coefficients etc.), and that we can faithfully retrieve such parameters and architectures using the mobster package.

### Subclonal deconvolution from the PD4120a breast tumour

We discuss an example application to a WGS dataset of a real primary breast cancer; this dataset has been first discussed in [7] and also re-analyzed in our recent work [9]. The analysis that we present here reports the same overall conclusions but is more detailed than the one in [9], as it serves to show multiple functionalities of the mobster package.

This breast cancer sample (PD4120a) has been sequenced at very high-coverage (approximately 180×) and presents with $n=4643$ somatic single-nucleotide variants (SNVs) mapping to chromosome 3; this reduced dataset has been generated and quality-checked for one of our earlier works [12]. Chromosome 3 is diploid and therefore the VAF analysis of mutations mapping to that chromosome is analogous to using CCF values (here re-scaled in $[\mathrm{0,1}]$). This is true because for diploid regions the CCF computation from VAF is trivial, and corresponds just to a purity adjustment.

**Fig. 2:**
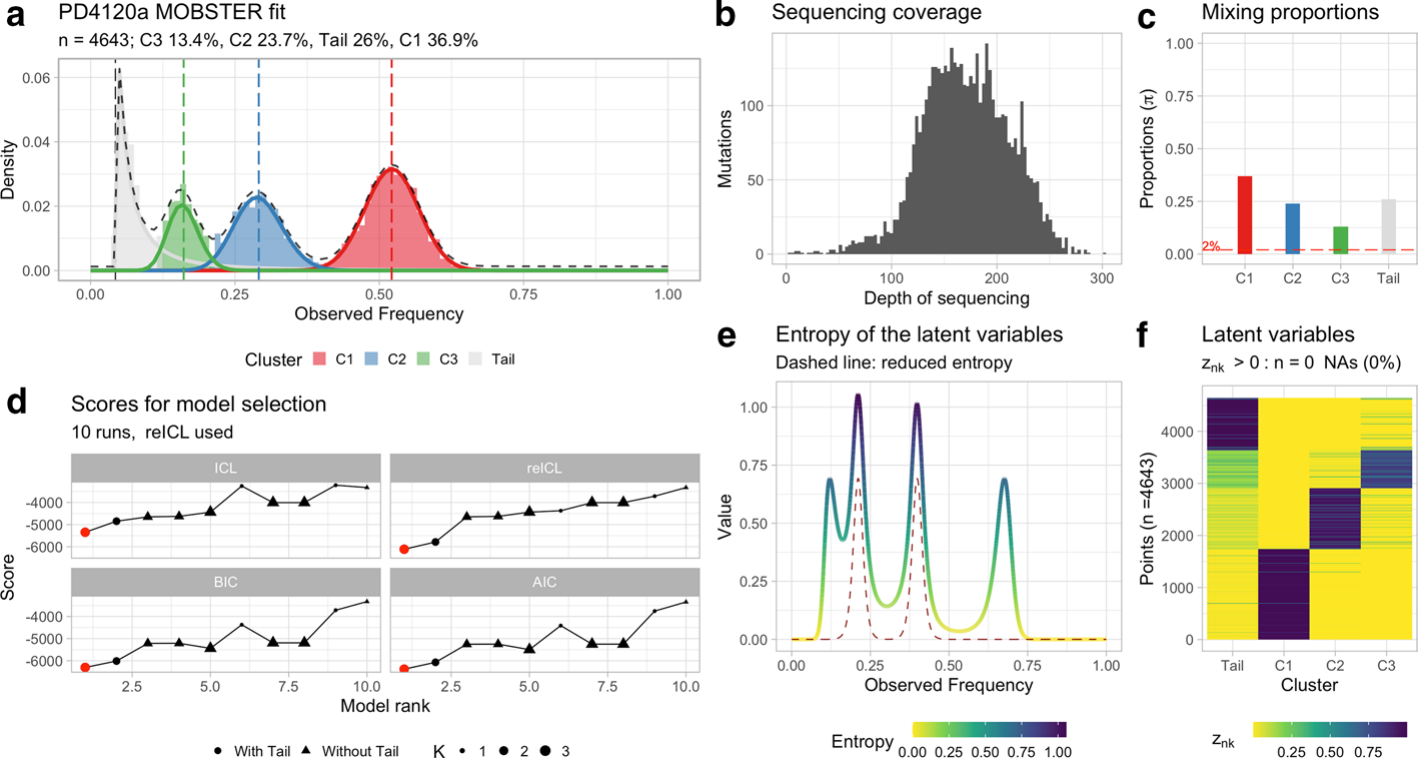
MOBSTER analysis of WGS sample PD4120a (breast cancer). **a** MOBSTER fit for $n=4643$ somatic mutations mapping to chromosome 3, which is largely diploid. Here the input VAF (Observed frequency) is adjusted by tumour purity. MOBSTER identifies $K=3$ Beta components and one tail, as shown previously in [CG20]. **b** Coverage for this sample as a histogram of the depth of sequencing for the input mutations. This sample has a median coverage of 169×. **c** Mixing proportions obtained from MOBSTER’s clustering assignments; these represent the proportion of mutations assigned to each one of the fit clusters in the model’s mixture. **d** Scores for model selection used by MOBSTER; in this case the model is selected by using the ICL score with reduced entropy, termed reICL. Note that all other scores suggest the same optimum model (red point in the score plot). This means that the identified model is the optimum no matter what scoring system we use. **e** Entropy of the model’s latent variables; we report both the standard entropy (solid line), as well as the reduced entropy which is computed just between mutations assigned to Beta clusters. As expected the reduced entropy is bounded from above by the standard entropy. **f** Value of the latent variable per mutation (cluster assignment probability). Here we assign via hard clustering assignments all mutations regardless the latent variables value. This shows that more uncertainty is found for mutations that map to clusters C3, C2 and Tail as the mixture density functions have overlapping support

A call to mobster_fit on the input mutations with default parameters computes the output model, as well as a number of alternative fitting solutions. The computation takes less than a couple of minutes on a standard laptop. The best output model computed using reICL is shown in Fig. 2a, while the sequencing depth of the input mutations is shown in Fig. 2b to show the high-quality of the input data. mobster fits the input data with $K=3$ Beta components, and one Pareto tail. This reflects a cancer sample that harbours clonal mutations, two distinct sub-clones enjoying a clonal expansion triggered by positive selection, and the tail of within-clone neutral dynamics. This 2-subclones model is a simplification of the v proposed in the original analysis of PD4120a [7]. As suggested in [9], the 2-subclones model seems more plausible in light of a complementary analysis carried out in [7], where it is shown that tail mutations after phasing associate to multiple nodes of the tumour’s clone tree. This is consistent with the signal of polyphyletic lineages that by definition constitute neutral mutations, and not with mutations hitchhiking tumour subclones. Therefore, the input $n=4643$ SNVs are assigned to 4 clusters by MOBSTER (Fig. 2c); the pool of clonal mutations represents the larger cluster (C1) with more than 30% of the input SNVs, the largest subclone (C2) and the tail have a similar number of mutations (i.e., about 25% of the input SNVs), and the smallest cluster is one of the two subclones (C3).

The package implements a number of distinct scores for model-selection, but the model in Fig. 2a is selected by using reICL. In this case all the scores available in the package rank the output model first, meaning that they consistently predict the model in Fig. 2a to be the best possible fit for the input data (Fig. 2d). The tool also offers visualization functions that can at least suggest, even from a single model fit, what is the stability of the clustering. This information is linked to model’s latent variables—denoted with ${\varvec{z}}$, and represented as a $n\times (k+1)$ matrix—which provide the probability of each of the input $n$ mutations to be assigned to the $k+1$ clusters ($k$ Beta plus one tail). In Fig. 2e we plot the entropy profile associated to each one of the input values in the domain $[\mathrm{0,1}]$; this is computed from the relative density of each mixture component in the model, and shows, for both the standard and reduced entropies, higher values where there is more uncertainty in clustering assignments. The full set of latent variables can also be visualized (Fig. 2f) as a heatmap, in this case highlighting more uncertainty for the assignments of the two subclones and the tail. This is expected since the three components largely overlap with similar density values.

**Fig. 3:**
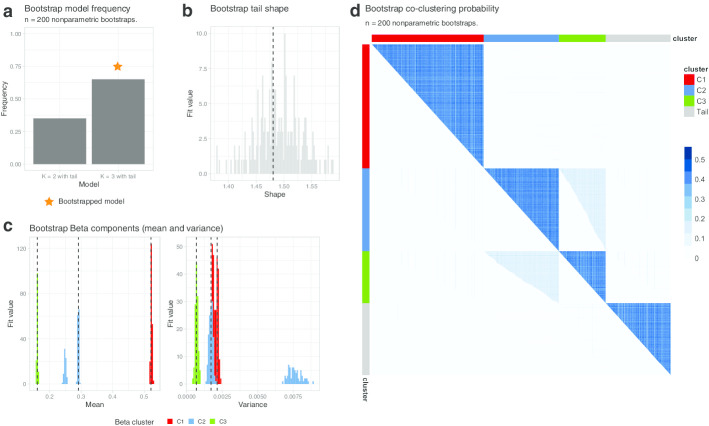
Analysis of the model in Fig. 2 using $n=200$ non-parametric bootstrap resamples. **a** Bootstrapped model frequency. The target model has $K=3$ Beta clusters, plus one tail. Across all the 200 resamples the target model is fit in 65% of the cases, and in the remaining 35% only two Beta components are used; in all cases the tail is always fit to data. **b **Bootstrapped distribution of the tail shape, with annotated point estimate (dashed line). **c** Bootstrapped distribution of the mean and variance of the Beta components, with annotated point estimate (dashed line). Note that cluster C2 can show bimodal distributions; this is due to the 35% bootstrapped cases in which C2 is the only subclonal cluster (i.e., C3 is dropped). Point estimates are annotated as dashed lines. **d** Co-clustering probability of each pair of points in the input data, visualized as a matrix ordered by the clustering assignments in Fig. 2. This shows that clonal mutations are very stable (C1), and that mutations in cluster C3 in a small proportion of cases are assigned together with mutations in cluster C2. This is confirmed by the plot in panel (**a**) where the model with $K=2$ Beta clusters plus one tail are identified in 35% of the non-parametric bootstrap resamples

Model confidence and stability can be formalized using bootstrap procedures. In Fig. 3 we show the result of a non-parametric bootstrap run with 200 resamples, which can be used to estimate bootstrap confidence intervals (CI) for all the model parameters at a desired $\alpha$-level (default 5%), and the overall model confidence. Figure 3a shows the overall model confidence; the selected output model of Fig. 2a is selected in 65% of the non-parametric bootstrap resamples, and in the remaining 35% of the cases a model with only $K=2$ Beta components and one tail is selected. In those models the signal lost is the one from the smallest subclone, C3, as one can immediately expect from Fig. 2 and the width of the Beta component C2 is increased to include SNVs originally assigned to C3. The 200 bootstrap resamples allow the estimation of a full distribution for the tail shape (CI [1.41–1.57]), as well as the Beta means and variance (means: C1 CI [0.51–0.52], C2 CI [0.24–0.29] and C3 CI [0.15–0.16], variance: C1 CI [0.001–0.002], C2 CI [0.001–0.008] and C3 CI [0.0005–0.0009]); the bootstrap distributions are plot in Fig. 3b, c. Notice that as expected the largest CIs are associated to C2, consistently to reflect the cases where cluster C3 is drop. The usage of a non-parametric bootstrap procedure allows also to estimate the co-clustering probability for each pair of mutations (Fig. 3f), which is bounded from above by the joint sampling probability for each pair of mutations (resampled with uniform probability in this bootstrap). This $n\times n$ matrix shows that the clonal cluster (C1) is extremely stable (dark blue gradient), and that lower stability involves the two subclonal clusters (C3 and C2), confirming the other assessments. On the overall, these metrics suggest that the quality of the fit computed with mobster, as measured from robustness of the overall model fit and its parameters, is very high and confirms that the breast cancer sample PD4120a likely contains two on-going subclonal expansions.

## Discussion

The mobster package implements the first statistical method that integrates Machine Learning and Population Genetics to perform tumour subclonal deconvolution from whole-genome DNA sequencing data of human cancers [9]. The method improves largely over standard methods for tumour deconvolution, as largely shown in [9], and is made accessible through the new R software package mobster which provides several functions for data pre-processing, visualization and analysis (model fitting, confidence assessment and post-clustering analysis). In this paper we described the principles underlying the R package and showed its analysis of one tumour simulated by a stochastic branching process model of tumour growth, and one polyclonal breast cancer sample. In the future we will extend the current package to support input/output with other common tools for cancer evolutionary analyses, such as callers for somatic mutations and copy number detection, and population-level inferences of patterns of tumour evolution from data of multiple patients.

## Supplementary information

**Additional file 1.** Supplementary notes that describe the software and its applications.

## Data Availability

The tool and data shown in this paper are available at the GitHub website hosting the mobster package: https://caravagnalab.github.io/mobster/. Project name: mobster. Project home page: https://caravagnalab.github.io/mobster/. Operating system(s): Platform independent. Programming language: R (version >= 3.6.0; with releases for both ~ 3.6 and > 4.0). Other requirements: open R packages (*ggplot2, sads, cli, clisymbols, cowplot, crayon, ctree, dndscv, dplyr, magrittr, reshape2, tidyr*) that are automatically installed by the tool. License: GNU GPL 3. Any restrictions to use by non-academics: none.

## Abbreviations

WGS: Whole-genome sequencing
VAF: Variant Allele Frequency
CCF: Cancer Cell Fraction
SNV: Single-nucleotide variant

## Footnotes

1 https://t-heide.github.io/TEMULATOR/
