# Supplementary material for: The MOBSTER R package for tumour subclonal deconvolution from bulk DNA whole-genome sequencing data
Source: BMC Bioinformatics. 2020 Nov 17;21:531. doi: 10.1186/s12859-020-03863-1 (PMC7672894; doi:10.1186/s12859-020-03863-1)

The mobster R package for tumour subclonal deconvolution from bulk DNA whole-genome sequencing data

Additional File 1

Giulio Caravagna*, Guido Sanguinetti, Trevor A Graham, Andrea Sottoriva*

November 9, 2020

Introduction

These notes describe most of the vignettes available at the mobster GitHub Pages website:

`https://caravagn.github.io/mobster/`.

Available notes cover the following topics:

- Note 1: introduction to the input format, simple fits and data-generation process;
- Note 2: plotting functions for the models and the data;
- Note 3: bootstrap estimation of the model parameters;
- Note 4: inference of population genetics parameters of tumour growth;
- Note 5: clone-specific dN/dS statistics;
- Note 6: clone tree estimation from the model fits.

The statistical model in mobster, and its first applications to real cancer data are described in:

- *Subclonal reconstruction of tumors using machine learning and population genetics.* Giulio Caravagna, Timon Heide, Marc Williams, Luis Zapata, Daniel Nichol, Ketevan Chkhaidze, William Cross, George D. Cresswell, Benjamin Werner, Ahmet Acar, Louis Chesler, Chris P. Barnes, Guido Sanguinetti, Trevor A. Graham, Andrea Sottoriva. Nature Genetics volume 52, pages 898-907 (2020),

*Correspondence at gcaravagna@units.it or andrea.sottoriva@icr.ac.uk

Note 1. Introduction: input formats, data generation and fits

```r
library(mobster)
library(tidyr)
library(dplyr)
```

1 Input data for `mobster`

You can run a MOBSTER analysis if, for a set of input mutations (SNVs, indels etc.), you have available VAF or CCF data. The input data can be loaded using different input formats.

For VAF values you can use:

- a `data.frame` (or, `tibble`) with a column named `VAF` whose values are numerical $0 < x < 1$, without `NA` entries;
- a VCF file that must contain at least a column with the total depth of sequencing and the number of reads with the variants. In this case you need first to use function `load_VCF` and load the VCF content, and then proceed using your data as a `data.frame`.

For CCF values you can only use the a `data.frame` format. Importantly, you have to store the CCF values in a column again named `VAF`, which must follow the same convention of a VAF column (i.e., range of values). Since CCF values usually peak at around `1.0` for clonal mutations (i.e., present in 100% of the input cells), we suggest to adjust standard CCF estimates dividing them by `0.5` in order to reflect the peak of an *heterozygous clonal mutation* at 50% VAF for a 100% pure bulk sample.

Example dataset. Diploid mutations from sample `LU4` of the Comprehensive Omics Archive of Lung Adenocarcinoma are available in the package under the name `LU4_lung_sample`. The available object is the results of an analysis with `mobster`, and the input mutation data is stored inside the object.

```r
# Example dataset LU4_lung_sample, downloaded from http://genome.kaist.ac.kr/
print(mobster::LU4_lung_sample$best$data)
#> # A tibble: 1,282 x 12
#>      Key Callers t_alt_count t_ref_count Variant_Classif~    DP    VAF chr
#>    <int> <chr>         <int>       <int> <chr>            <int>  <dbl> <chr>
#>  1   370 mutect~          30          95 intergenic         125 0.24   chr2
#>  2   371 mutect~          10         105 intergenic         115 0.0870 chr2
#>  3   372 mutect~          40         108 intronic           148 0.270  chr2
#>  4   373 mutect~          20         101 intronic           121 0.165  chr2
#>  5   374 mutect~          39          89 intergenic         128 0.305  chr2
#>  6   375 mutect~          41         120 intronic           161 0.255  chr2
#>  7   376 mutect~          43          93 intronic           136 0.316  chr2
#>  8   377 mutect~          22          95 intergenic         117 0.188  chr2
#>  9   378 mutect            7         104 intergenic         111 0.0631 chr2
#> 10   379 mutect~          35          93 intergenic         128 0.273  chr2
#> # ... with 1,272 more rows, and 4 more variables: from <chr>, ref <chr>,
#> #   alt <chr>, cluster <chr>
```

Other datasets are available through the `data` command.

1.1 Driver annotations

In the context of subclonal deconvolution we are often interested in linking "driver" events to clonal expansions. Since `mobster` works with somatic mutations data, it is possible to annotate the status of "driver mutation" in the input data; doing so, the drivers will be reported in some visualisations of the tool, but will not influence any of the computation carried out in `mobster`.

The annotate one or more *driver mutations* you need to include in your column 2 extra columns:

- `is_driver`, a boolean `TRUE/FALSE` flag;
- `driver_label`, a character string that will be used as label in any visualisation that uses drivers.

2 Generating random models and data

You can sample a random dataset with the `random_dataset` function, setting:

- the number of mutations (`n`) and Beta components (`k`, subclones) to generate;
- ranges and constraints on the size of the components;
- ranges for the mean and variance of the Beta components.

The variance of the Betas is defined as u/B where $u \sim U[0,1]$, and B is the input parameter `Beta_variance_scaling`. Roughly, values of `Beta_variance_scaling` on the order of `1000` give low variance and sharp peaked data distributions. Values on the order of `100` give much wider distributions.

```
dataset = random_dataset(
  seed = 123456789,
  Beta_variance_scaling = 100    # variance ~ U[0, 1]/Beta_variance_scaling
  )
```

A list with 3 components is returned, which contains the actual data, sampled parameters of the generative model, and a plot of the data.

In `mobster` we provide the implementation of the model's density function (`ddbpmm`, density Dirichlet Beta Pareto mixture model), and a sampler (`rdbpmm`) which is used internally by `random_dataset` to generate the data.

```
# Data, in the MOBSTER input format with a "VAF" column.
print(dataset$data)
#> # A tibble: 5,000 x 2
#>      VAF cluster
#>    <dbl> <chr>
#>  1 0.856 C1
#>  2 0.853 C1
#>  3 0.844 C1
#>  4 0.827 C1
#>  5 0.855 C1
#>  6 0.854 C1
#>  7 0.878 C1
#>  8 0.865 C1
#>  9 0.845 C1
#> 10 0.874 C1
#> # ... with 4,990 more rows

# The generated model contains the parameters of the Beta components (a and b),
# the shape and scale of the tail, and the mixing proportion.
print(dataset$model)
```

```
#> $a
#>       C1      C2
#> 72.6286 30.5932
#>
#> $b
#>        C1       C2
#> 13.07363 24.93900
#>
#> $shape
#> [1] 1
#>
#> $scale
#> [1] 0.05
#>
#> $pi
#>      Tail        C1        C2
#> 0.3431634 0.3331162 0.3237204

# A plot object (ggplot) is available where each data-point is coloured by
# its generative mixture component. The vertical lines annontate the means of
# the sampled Beta distributions.
print(dataset$plot)
```

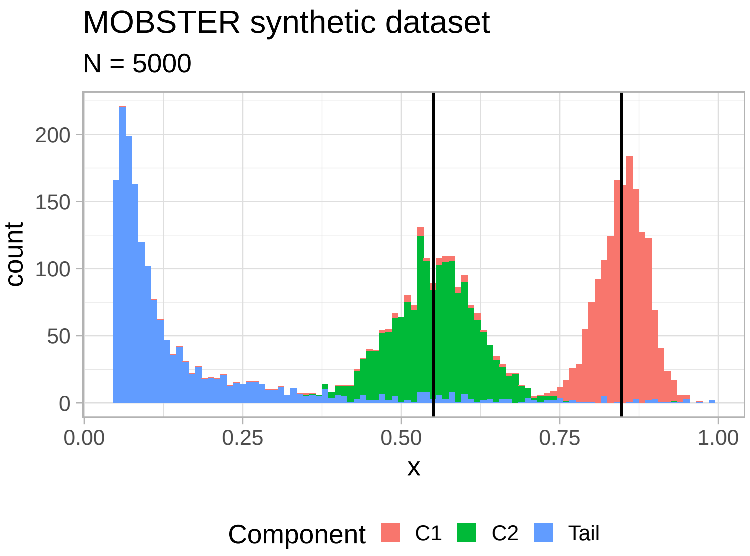

3 Fitting a dataset

Function `mobster_fit` fits a MOBSTER model.

The function implements a model-selection routine that by default scores models by their `reICL` (*reduced Integrative Classification Likelihood*) score, a variant to the popular `BIC` that uses the entropy of the latent variables of the mixture. `reICL` is discussed in the main paper.

This function has several parameters to customize the fitting procedure, and a set of special pre-parametrised runs that can be activated with parameter `auto_setup`. Here we use `auto_setup = "FAST"`, an automatic setup for a fast run; its parameters are accessible through an internal package function.

```
# Hidden function (:::)
mobster:::template_parameters_fast_setup()
#> $K
#> [1] 1 2
#>
#> $samples
#> [1] 2
#>
#> $init
#> [1] "random"
#>
#> $tail
#> [1]  TRUE FALSE
#>
#> $epsilon
#> [1] 1e-06
#>
#> $maxIter
#> [1] 100
#>
#> $fit.type
#> [1] "MM"
#>
#> $seed
#> [1] 12345
#>
#> $model.selection
#> [1] "reICL"
#>
#> $trace
#> [1] FALSE
#>
#> $parallel
#> [1] FALSE
#>
#> $pi_cutoff
#> [1] 0.02
#>
#> $N_cutoff
#> [1] 10
```

Compared to these, default parameters test more extensive types of fits (i.e., more clones, longer fits, higher number of replicates etc.). We usually use the fast parametrisation to obtain a first fit of the data and, if not satisfied, we run customised calls of `mobster_fit`.

```
# Fast run with auto_setup = "FAST"
fit = mobster_fit(
  dataset$data,
  auto_setup = "FAST"
  )
#>  [ MOBSTER fit ]
#>  Loaded input data, n = 5000.
#>  n = 5000. Mixture with k = 1,2 Beta(s). Pareto tail: TRUE and FALSE. Output clusters with
#>  > 0.02 and n > 10.
```

```
#> ! mobster automatic setup FAST for the analysis.
#>   Scoring (without parallel) 2 x 2 x 2 = 8 models by reICL.
#>   MOBSTER fits completed in 10.1s.
#> -- [ MOBSTER ] My MOBSTER model n = 5000 with k = 2 Beta(s) and a tail ----------------------
#>   Clusters:   = 35% [C2], 33% [Tail] and 32% [C1], with   > 0.
#>   Tail [n = 1567, 33%] with alpha = 1.3.
#>   Beta C1 [n = 1636, 32%] with mean = 0.85.
#>   Beta C2 [n = 1797, 35%] with mean = 0.55.
#>   Score(s): NLL = -2558.66; ICL = -4657.61 (-5009.64), H = 383.05 (31.02). Fit converged by
#> MM in 28 steps.
```

A call of `mobster_fit` will return a list with 3 elements:

- the best fit `fit$best`, according to the selected scoring method;
- `fit$runs`, a list with the ranked fits; `best` matches the head of this list;
- `fit$fits.table`, a table that summarises the scores for each one of the runs.

Each fit object (`best` or any object stored in `runs`) is from the S3 class `dbpmm`.

```
# Print the best model
print(fit$best)
#> -- [ MOBSTER ] My MOBSTER model n = 5000 with k = 2 Beta(s) and a tail ----------------------
#>   Clusters:   = 35% [C2], 33% [Tail] and 32% [C1], with   > 0.
#>   Tail [n = 1567, 33%] with alpha = 1.3.
#>   Beta C1 [n = 1636, 32%] with mean = 0.85.
#>   Beta C2 [n = 1797, 35%] with mean = 0.55.
#>   Score(s): NLL = -2558.66; ICL = -4657.61 (-5009.64), H = 383.05 (31.02). Fit converged by
#> MM in 28 steps.

# Print top-3 models
print(fit$runs[[1]])
#> -- [ MOBSTER ] My MOBSTER model n = 5000 with k = 2 Beta(s) and a tail ----------------------
#>   Clusters:   = 35% [C2], 33% [Tail] and 32% [C1], with   > 0.
#>   Tail [n = 1567, 33%] with alpha = 1.3.
#>   Beta C1 [n = 1636, 32%] with mean = 0.85.
#>   Beta C2 [n = 1797, 35%] with mean = 0.55.
#>   Score(s): NLL = -2558.66; ICL = -4657.61 (-5009.64), H = 383.05 (31.02). Fit converged by
#> MM in 28 steps.
print(fit$runs[[2]])
#> -- [ MOBSTER ] My MOBSTER model n = 5000 with k = 1 Beta(s) and a tail ----------------------
#>   Clusters:   = 71% [C1] and 29% [Tail], with   > 0.
#>   Tail [n = 1429, 29%] with alpha = 1.5.
#>   Beta C1 [n = 3571, 71%] with mean = 0.68.
#>   Score(s): NLL = -1446.64; ICL = -2379.16 (-2842.18), H = 463.01 (0). Fit converged by MM
#> in 22 steps.
print(fit$runs[[3]])
#> -- [ MOBSTER ] My MOBSTER model n = 5000 with k = 1 Beta(s) and a tail ----------------------
#>   Clusters:   = 71% [C1] and 29% [Tail], with   > 0.
#>   Tail [n = 1429, 29%] with alpha = 1.5.
#>   Beta C1 [n = 3571, 71%] with mean = 0.68.
#>   Score(s): NLL = -1446.64; ICL = -2379.17 (-2842.18), H = 463.01 (0). Fit converged by MM
#> in 22 steps.
```

Usually, one keeps working with the `best` model fit. From that it is possible to extract the results of the fit, and the clustering assignments. The output is a copy of the input data, with a column reporting the model's

latent variables (LVs) and the `cluster` assignment (*hard clustering*).

```
# All assignments
Clusters(fit$best)
#> # A tibble: 5,000 x 5
#>      VAF cluster    Tail    C1           C2
#>    <dbl> <chr>     <dbl> <dbl>        <dbl>
#>  1 0.856 C1      0.00371 0.996 0.000000547
#>  2 0.853 C1      0.00375 0.996 0.000000759
#>  3 0.844 C1      0.00403 0.996 0.00000242
#>  4 0.827 C1      0.00520 0.995 0.0000187
#>  5 0.855 C1      0.00371 0.996 0.000000571
#>  6 0.854 C1      0.00373 0.996 0.000000684
#>  7 0.878 C1      0.00415 0.996 0.0000000246
#>  8 0.865 C1      0.00370 0.996 0.000000171
#>  9 0.845 C1      0.00398 0.996 0.00000208
#> 10 0.874 C1      0.00392 0.996 0.0000000480
#> # ... with 4,990 more rows

# Assignments with LVs probability above 85%
Clusters(fit$best, cutoff_assignment = 0.85)
#> # A tibble: 5,000 x 5
#>      VAF cluster    Tail    C1           C2
#>    <dbl> <chr>     <dbl> <dbl>        <dbl>
#>  1 0.856 C1      0.00371 0.996 0.000000547
#>  2 0.853 C1      0.00375 0.996 0.000000759
#>  3 0.844 C1      0.00403 0.996 0.00000242
#>  4 0.827 C1      0.00520 0.995 0.0000187
#>  5 0.855 C1      0.00371 0.996 0.000000571
#>  6 0.854 C1      0.00373 0.996 0.000000684
#>  7 0.878 C1      0.00415 0.996 0.0000000246
#>  8 0.865 C1      0.00370 0.996 0.000000171
#>  9 0.845 C1      0.00398 0.996 0.00000208
#> 10 0.874 C1      0.00392 0.996 0.0000000480
#> # ... with 4,990 more rows
```

The second call imposes a cut to the assignments with less than 85% probability mass in the LVs.

If you want to assign some new data to the fit model you can use function `Clusters_denovo`.

4 Basic plots of a fit

Clusters can be plot as an histogram with the model density (total and per mixture). By default, `mobster` names Beta clusters `C1`, `C2`, etc. according to the decreasing order of their mean; so `C1` is always the cluster with highest Beta mean, etc. If the data are diploid mutations, `C1` should represent clonal mutations.

```
# Plot the best model
plot(fit$best)
```

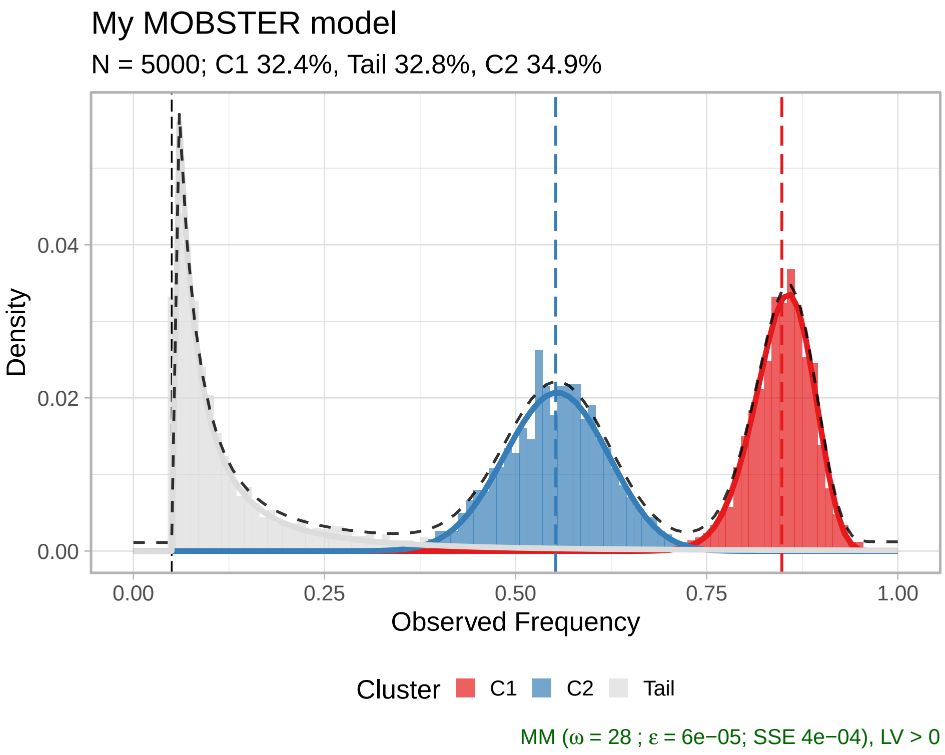

A comparative plot between the fit and data is assembled using cowplot.

```
cowplot::plot_grid(
  dataset$plot,
  plot(fit$best),
  ncol = 2,
  align = 'h')
```

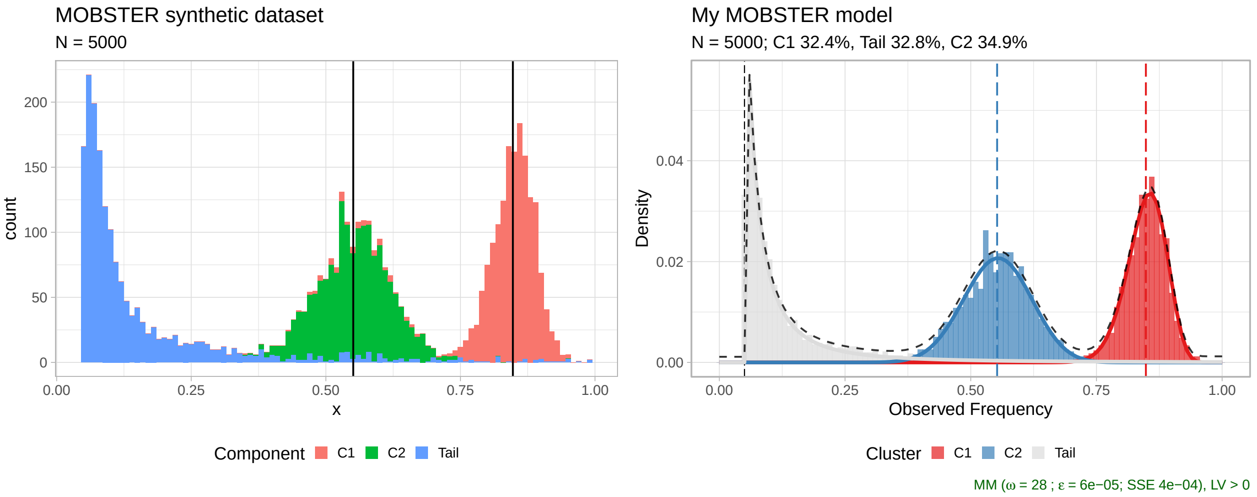

Note 2. Plotting models, data and fit parameters

```r
library(mobster)
library(tidyr)
library(dplyr)
```

This vignette describes the plotting functions available in `mobster`. As an example, we use one of the available datasets where we annotate some random mutations as drivers.

```r
# Example data where we have 3 events as drivers
example_data = Clusters(mobster::fit_example$best)

# Drivers annotation (we selected this entries to have nice plots)
drivers_rows = c(2239, 3246, 3800)

example_data$is_driver = FALSE
example_data$driver_label = NA

example_data$is_driver[drivers_rows] = TRUE
example_data$driver_label[drivers_rows] = c("DR1", "DR2", "DR3")

# Fit and print the data
fit = mobster_fit(example_data, auto_setup = 'FAST')
#>  [ MOBSTER fit ]
#>   Loaded input data, n = 5000.
#>   n = 5000. Mixture with k = 1,2 Beta(s). Pareto tail: TRUE and FALSE. Output clusters with
#>   > 0.02 and n > 10.
#> ! mobster automatic setup FAST for the analysis.
#>   Scoring (without parallel) 2 x 2 x 2 = 8 models by reICL.
#>   MOBSTER fits completed in 7.3s.
#> -- [ MOBSTER ] My MOBSTER model n = 5000 with k = 2 Beta(s) and a tail ----------------------
#>   Clusters:   = 55% [C1], 31% [Tail] and 14% [C2], with   > 0.
#>   Tail [n = 1370, 31%] with alpha = 1.2.
#>   Beta C1 [n = 2784, 55%] with mean = 0.48.
#>   Beta C2 [n = 846, 14%] with mean = 0.15.
#>   Score(s): NLL = -5671.5; ICL = -10359.09 (-11266.35), H = 907.26 (0). Fit converged by MM
#> in 75 steps.
#>   The fit object model contains also drivers annotated.
#> # A tibble: 3 x 7
#>      VAF cluster   Tail       C1       C2 is_driver driver_label
#>    <dbl> <chr>    <dbl>    <dbl>    <dbl> <lgl>     <chr>
#> 1 0.448  C1      0.0125 9.88e- 1 8.08e-21 TRUE      DR1
#> 2 0.159  C2      0.225  2.35e-34 7.75e- 1 TRUE      DR2
#> 3 0.0629 Tail    1.00   1.91e-82 4.02e- 5 TRUE      DR3

best_fit = fit$best
print(best_fit)
#> -- [ MOBSTER ] My MOBSTER model n = 5000 with k = 2 Beta(s) and a tail ----------------------
#>   Clusters:   = 55% [C1], 31% [Tail] and 14% [C2], with   > 0.
```

```
#>   Tail [n = 1370, 31%] with alpha = 1.2.
#>   Beta C1 [n = 2784, 55%] with mean = 0.48.
#>   Beta C2 [n = 846, 14%] with mean = 0.15.
#>   Score(s): NLL = -5671.5; ICL = -10359.09 (-11266.35), H = 907.26 (0). Fit converged by MM
#> in 75 steps.
#>   The fit object model contains also drivers annotated.
#> # A tibble: 3 x 7
#>      VAF cluster   Tail       C1       C2 is_driver driver_label
#>    <dbl> <chr>    <dbl>    <dbl>    <dbl> <lgl>     <chr>
#> 1 0.448  C1      0.0125 9.88e- 1 8.08e-21 TRUE      DR1
#> 2 0.159  C2      0.225  2.35e-34 7.75e- 1 TRUE      DR2
#> 3 0.0629 Tail    1.00   1.91e-82 4.02e- 5 TRUE      DR3
```

1 Model plots

The plot reports some fit statistics, and shows the annotated drivers if any.

```
plot(best_fit)
```

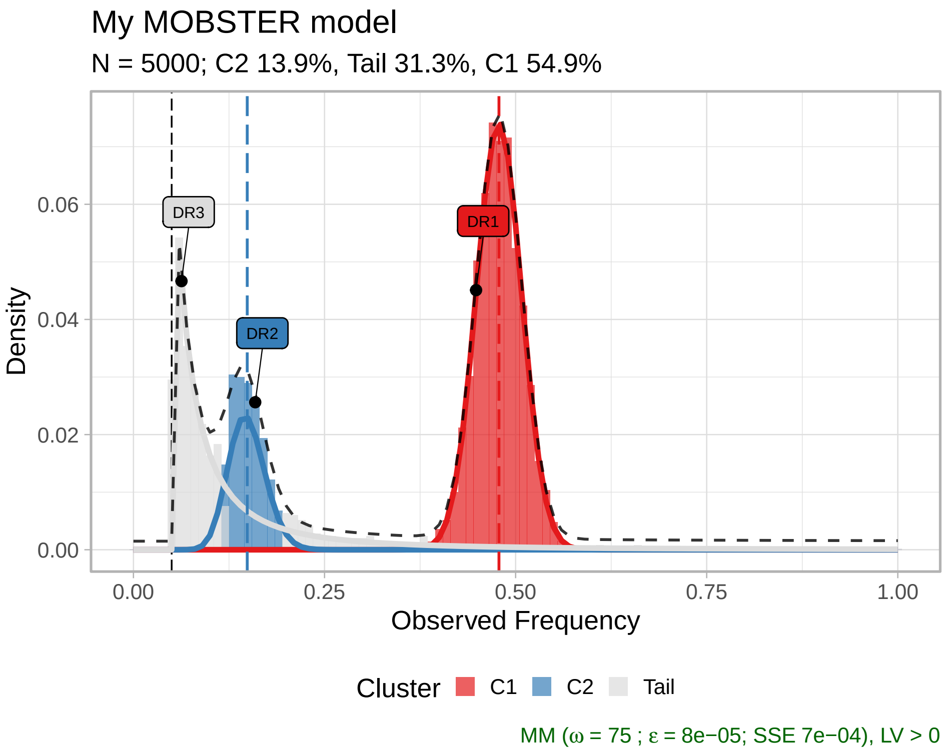

One can hide the drivers setting `is_driver` to `FALSE`.

```
copy_best_fit = best_fit
copy_best_fit$data$is_driver = FALSE

plot(copy_best_fit)
```

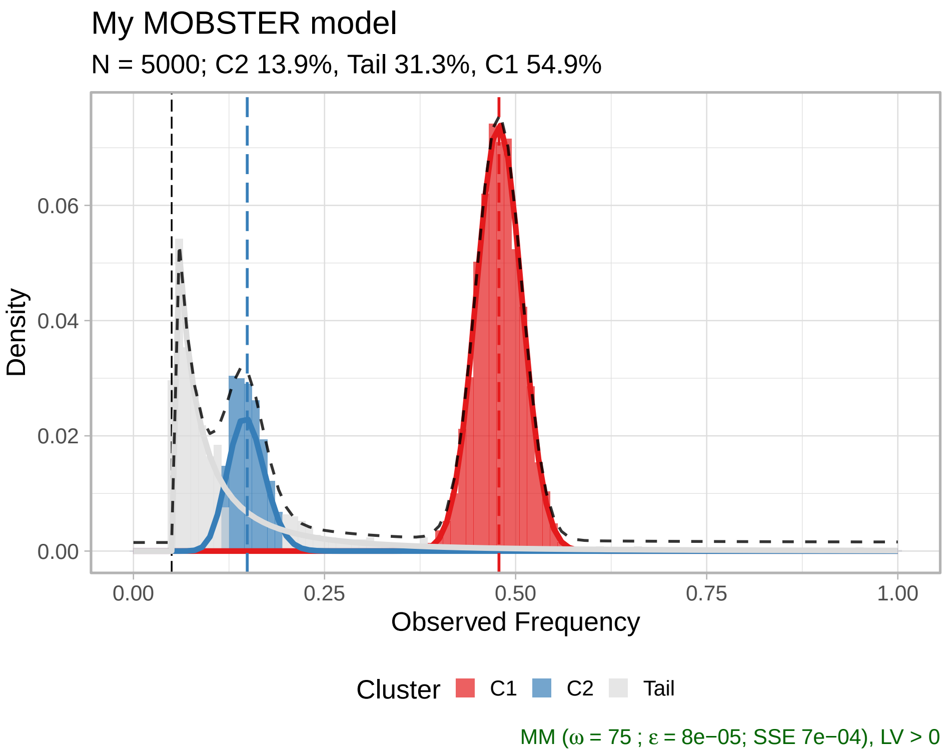

It is possible to annotate further labels to this plot, providing just a VAF value and a `driver_label` value. Each annotation points to the VAF value on the x-axis, and on the corresponding mixture density value for the y-axis.

```
plot(best_fit,
     annotation_extras =
       data.frame(
         VAF = .35,
         driver_label = "Something",
         stringsAsFactors = FALSE)
     )
```

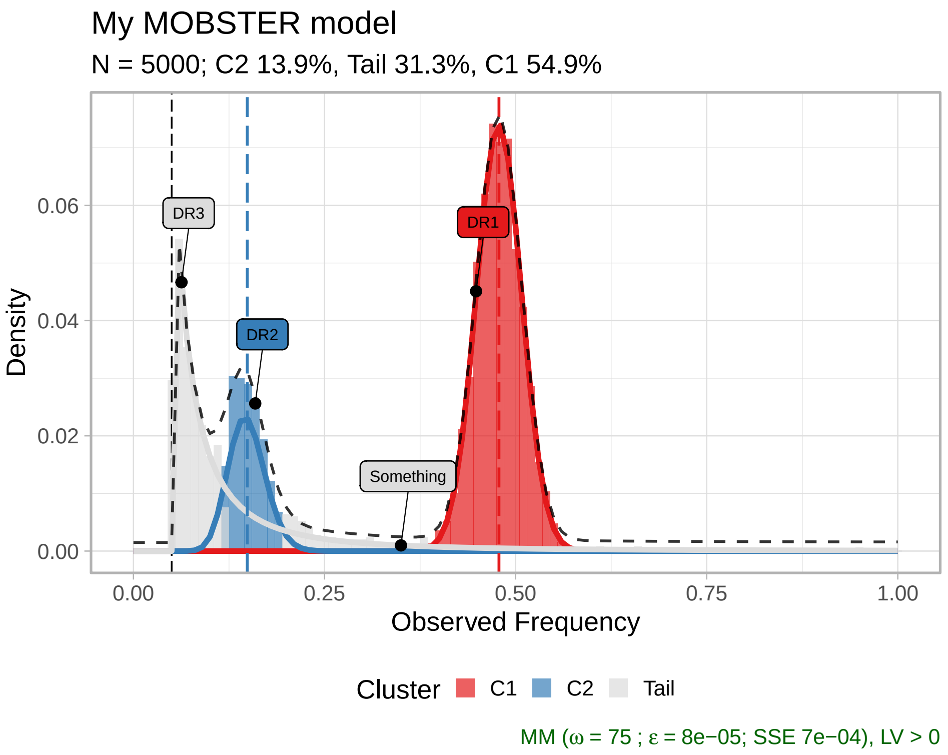

Other visualisations can be obtained as follows

```
ggpubr::ggarrange(
  plot(best_fit, alpha = .3),                        # Histogram transparency
  plot(best_fit, colors = c(`Tail` = 'darkorange')), # Tail color
  plot(best_fit, cutoff_assignment = .95),           # Hide mutations based on latent variables (see be
  plot(best_fit, secondary_axis = "SSE"),            # Add a mirrored y-axis with the % of SSE
  ncol = 2,
  nrow = 2
)
```

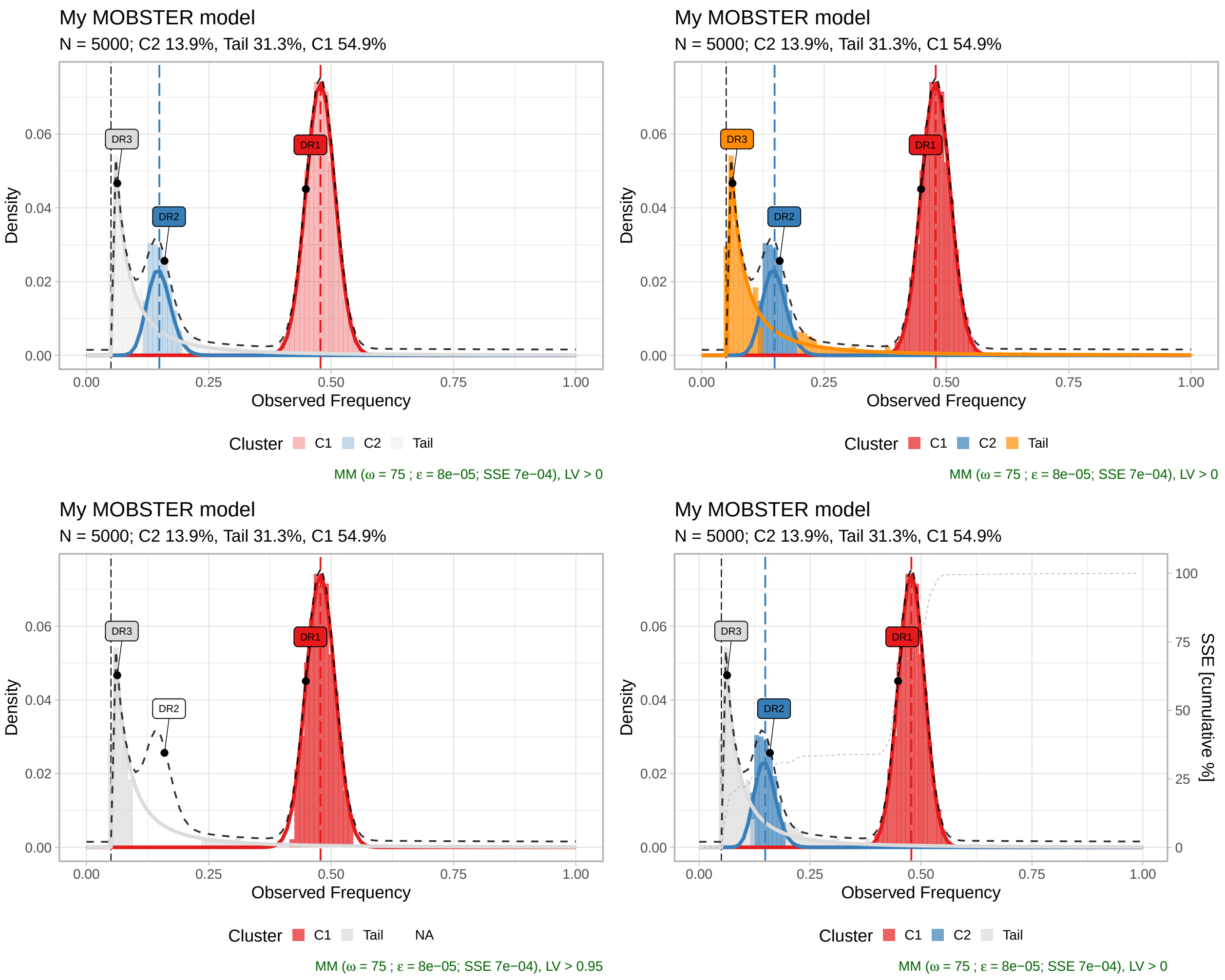

2 Fit statistics

You can plot the latent variables, which are used to determine *hard clustering* assignments of the input mutations. A `cutoff_assignment` determines a cut to prioritize assignments above the hard clustering assignments probability. Non-assignable mutations (`NA` values) are on top of the heatmap.

```
ggpubr::ggarrange(
  mobster::plot_latent_variables(best_fit, cutoff_assignment = 0),
  mobster::plot_latent_variables(best_fit, cutoff_assignment = 0.4),
  mobster::plot_latent_variables(best_fit, cutoff_assignment = 0.8),
  mobster::plot_latent_variables(best_fit, cutoff_assignment = 0.97),
  ncol = 4,
  nrow = 1
)
```

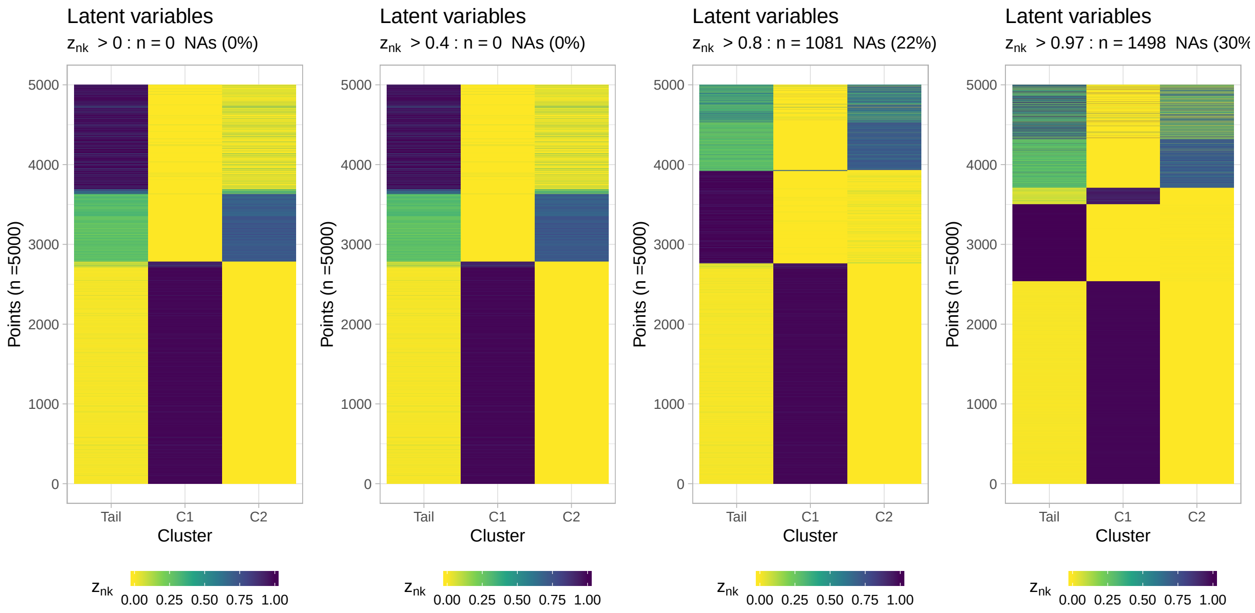

You can plot a barplot of the mixing proportions, using the same colour scheme for the fit (here, default). The barplot annotates a dashed line by default at 2%.

```r
plot_mixing_proportions(best_fit)
```

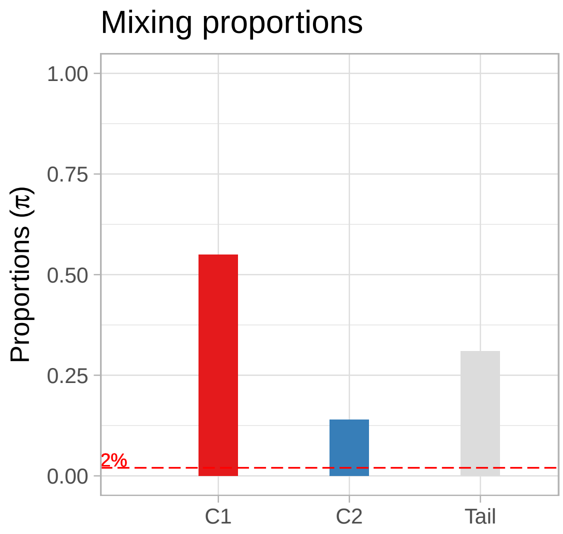

The negative log-likelihood `NLL` of the fit can be plot against the iteration steps, so to check that the trend is decreasing over time.

```r
plot_NLL(best_fit)
```

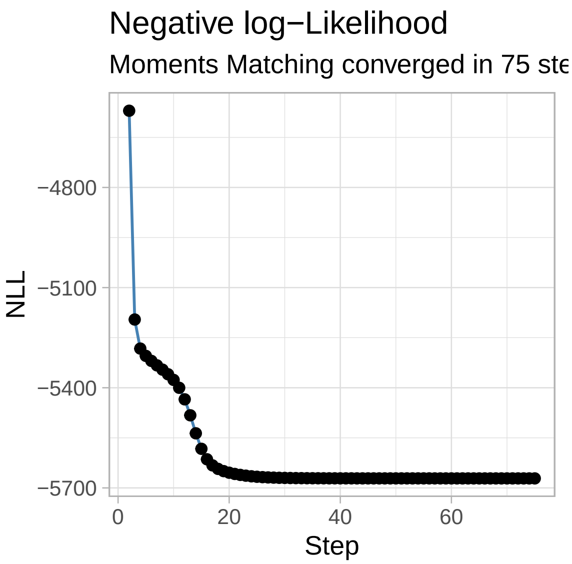

A contributor to the NLL is the entropy of the mixture, which can be visualized along with the reduced entropy, which is used by reICL.

```
plot_entropy(best_fit)
```

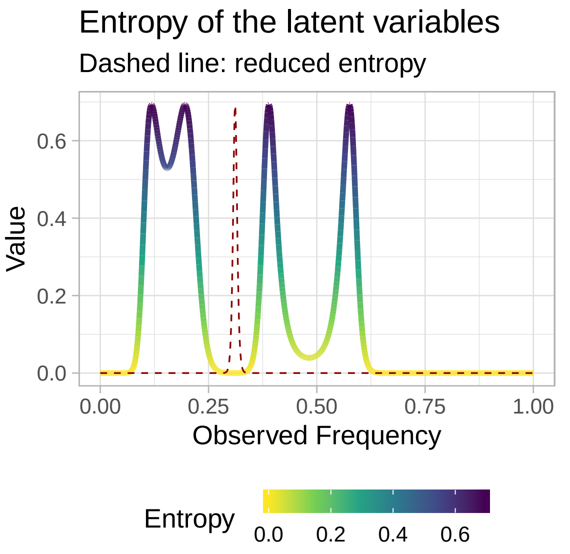

3 Inspecting alternative fits

Alternative models are returned by function mobster_fit, and can be easly visualized; the table reporting the scores is a good place to start investigating alternative fits to the data.

```
print(fit$fits.table)
#>          NLL        BIC        AIC   entropy        ICL reduced.entropy
#> 5 -5671.504 -11266.353 -11325.008  907.2581 -10359.095    8.466073e-11
#> 1 -5332.989 -10614.876 -10653.979  323.0130 -10291.863    0.000000e+00
#> 2 -5332.968 -10589.280 -10647.935 2186.1915  -8403.089    1.903207e+03
#> 4 -4442.905  -8834.707  -8873.810  544.0481  -8290.658    5.440481e+02
#> 3 -1570.709  -3115.867  -3135.419    0.0000  -3115.867    0.000000e+00
#>         reICL size K  tail
```

```
#> 5 -11266.353    9 2  TRUE
#> 1 -10614.876    6 1  TRUE
#> 2  -8686.073    9 2  TRUE
#> 4  -8290.658    6 2 FALSE
#> 3  -3115.867    3 1 FALSE
```

Top three fits, plot in-line.

```
ggpubr::ggarrange(
  plot(fit$runs[[1]]),
  plot(fit$runs[[2]]),
  plot(fit$runs[[3]]),
  ncol = 3,
  nrow = 1
)
```

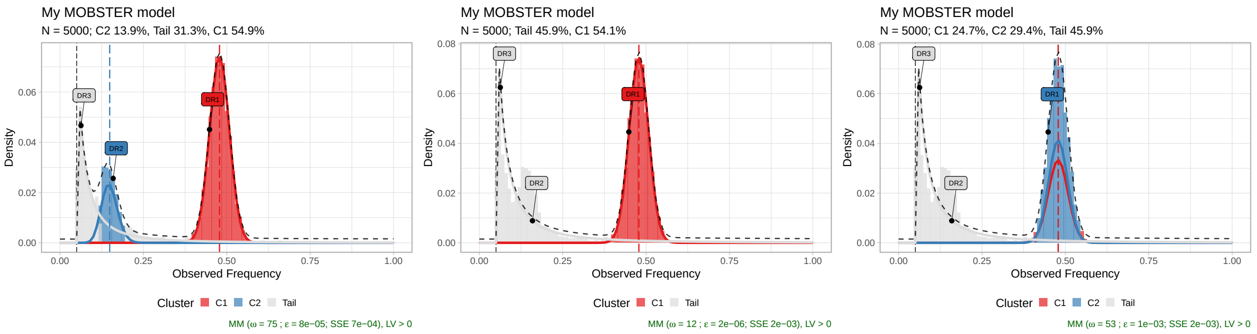

The sum of squared error (SSE) between the fit density and the data (binned with bins of size `0.01`), can be plot to compare multiple fits. This measure is a sort of "goodness of fit" statistics.

```
# Goodness of fit (SSE), for the top 3 fits.
plot_gofit(fit, TOP = 3)
```

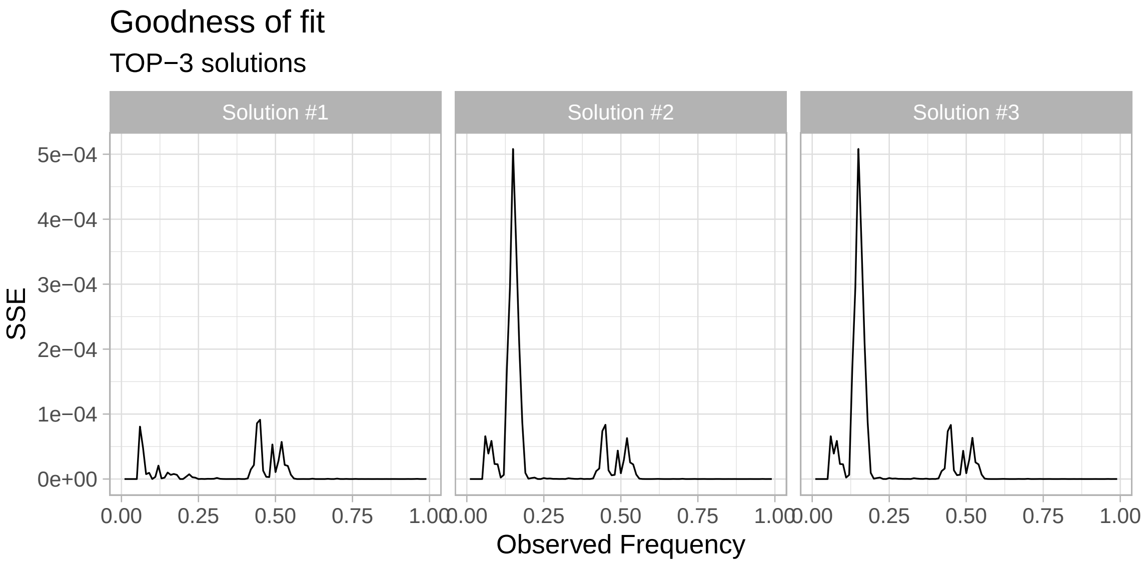

The scores for model selection can also be compared graphically.

```
plot_fit_scores(fit)
```

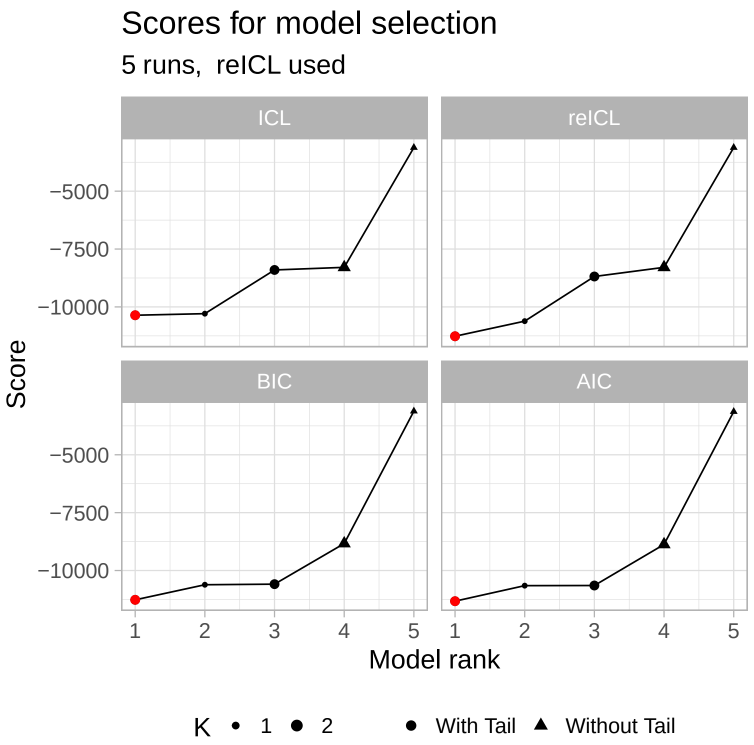

In the above plot, all the computed scoring functions (`BIC`, `AIC`, `ICL` and `reICL`) are shown. This plot can be used to quickly grasp the best model with, for instance, the default score (`reICL`) is also the best for other scores. In this graphics the red dot represents the best model according to each possible score.

4 Model-selection report

A general model-selection report assembles most of the above graphics.

```
plot_model_selection(fit, TOP = 5)
```

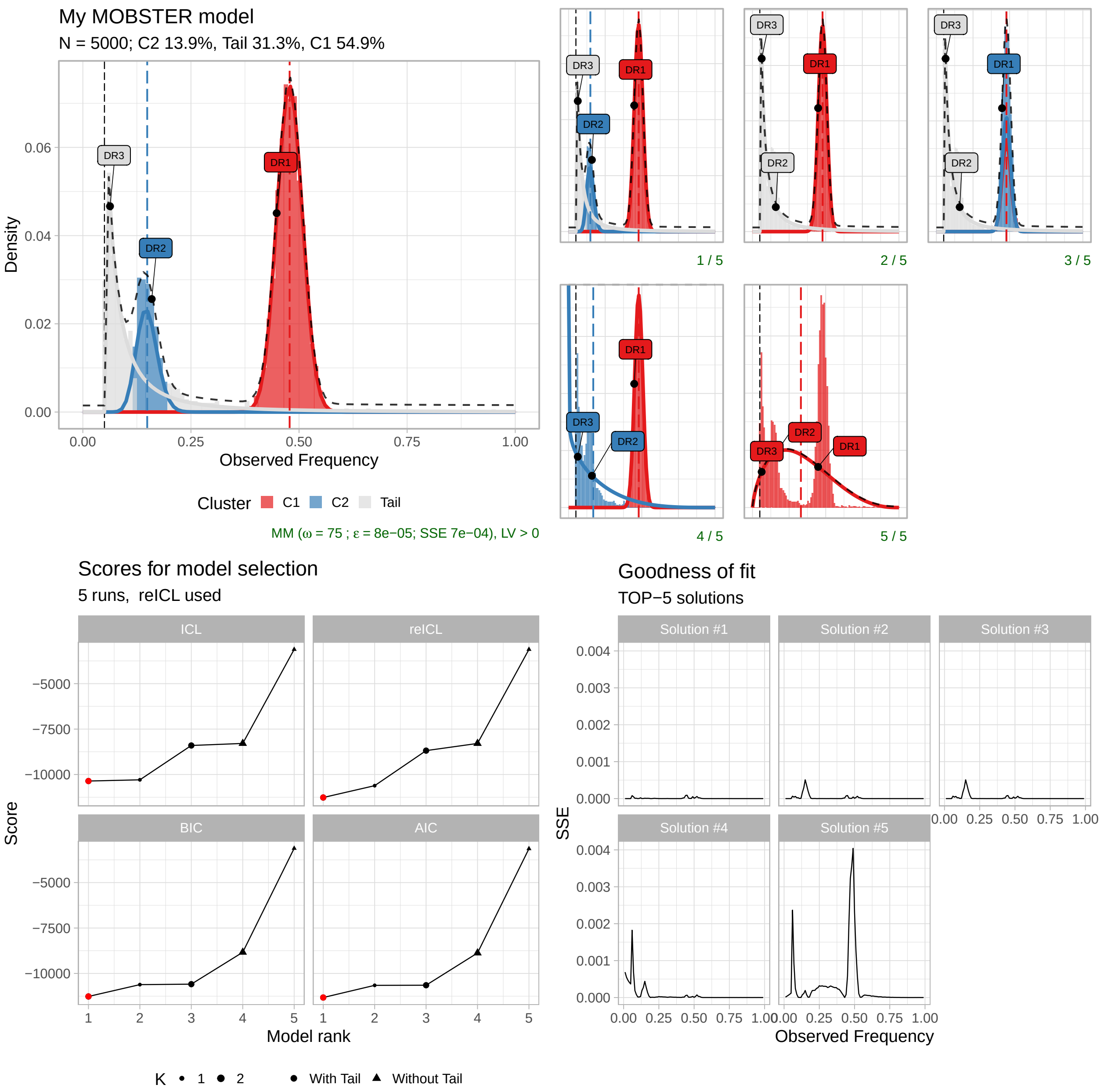
My MOBSTER model
N = 5000; C2 13.9%, Tail 31.3%, C1 54.9%
Density
Observed Frequency
DR1
DR2
DR3
Cluster C1 C2 Tail
MM (ω = 75 ; ε = 8e−05; SSE 7e−04), LV > 0
1 / 5
2 / 5
3 / 5
4 / 5
5 / 5
Scores for model selection
5 runs, reICL used
ICL
reICL
BIC
AIC
Score
Model rank
K 1 2 With Tail Without Tail
Goodness of fit
TOP−5 solutions
Solution #1
Solution #2
Solution #3
Solution #4
Solution #5
SSE
Observed Frequency

Note 3. Model and parameters confidence estimation by the bootstrap

```
library(mobster)
library(tidyr)
library(dplyr)
```

1 Bootstrapping a model

This vignette describes how to compute the *bootstrap* confidence of a MOBSTER model.

Both *parametric* and *nonparametric* bootstrap options are available: the former samples data from the model, the latter re-samples the data (with repetitions). Statistics are bootstrap estimates (averages) of the bootstrap fits. In both cases a model bootstrap probability can be computed, as well as the probability of clustering together any two mutations.

We show this with a small synthetic dataset .to speed up the computation.

```
# Data generation
dataset = random_dataset(
  N = 400,
  seed = 123,
  Beta_variance_scaling = 100
  )

# Fit model -- FAST option to speed up the vignette
fit = mobster_fit(dataset$data, auto_setup = 'FAST')
#>  [ MOBSTER fit ]

# Composition with cowplot
cowplot::plot_grid(
  dataset$plot,
  plot(fit$best),
  ncol = 2,
  align = 'h') %>%
  print
```

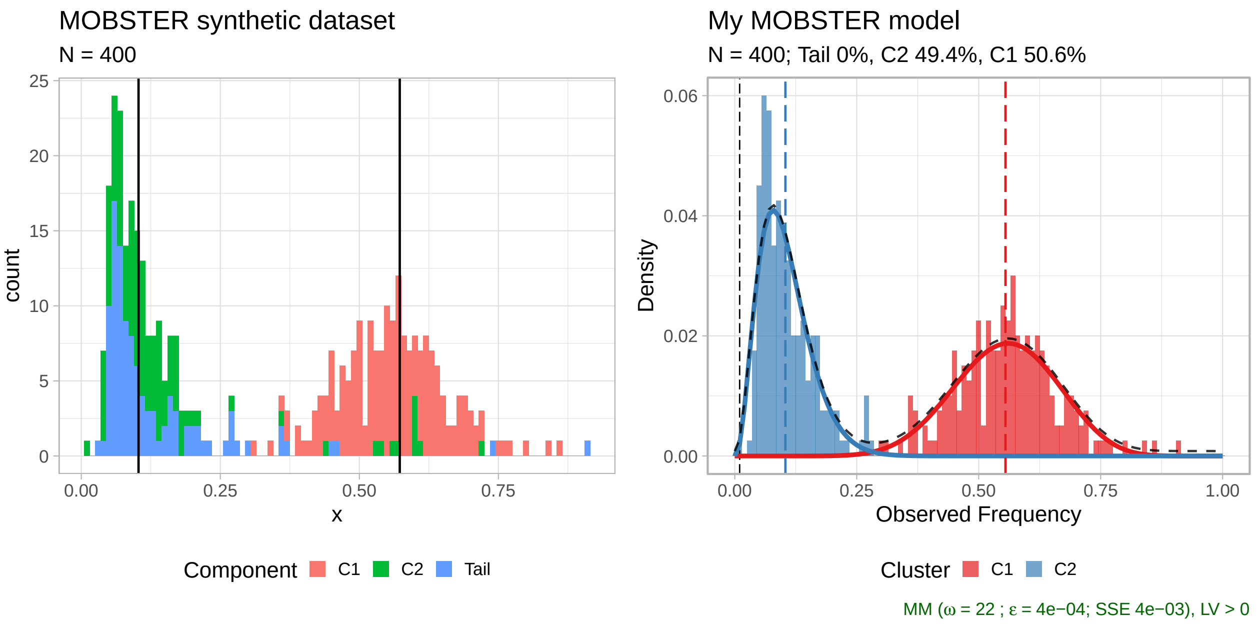

Now we can compute `n.resamples` nonparametric bootstraps using function `mobster_bootstrap`, passing parameters to the calls of `mobster_fit`. This function by defaults runs the fits in parallel (using a default percentage of the available cores); parallel computing capabilities are achieved using package easypar.

```
# The returned object contains also the list of bootstrap resamples, and the fits.
bootstrap_results = mobster_bootstrap(
  fit$best,
  bootstrap = 'nonparametric',
  n.resamples = 25,
  auto_setup = 'FAST' # forwarded to mobster_fit
  )
#>  [ MOBSTER bootstrap ~ 25 resamples from nonparametric bootstrap ]
```

The output object includes the bootstrap resamples, the fits and possible error returned by the runs.

```
# Resamples are available for inspection as list of lists,
# with a mapping to record the mutation id of the resample data.
# Ids are row numbers.
print(bootstrap_results$resamples[[1]][[1]] %>% as_tibble())
#> # A tibble: 400 x 3
#>        id    VAF original.id
#>     <int>  <dbl>       <int>
#>  1      1 0.623           93
#>  2      2 0.608          189
#>  3      3 0.564           13
#>  4      4 0.515          175
#>  5      5 0.127          207
#>  6      6 0.515          175
#>  7      7 0.560           79
#>  8      8 0.111          240
#>  9      9 0.388           62
#> 10     10 0.0669         375
#> # ... with 390 more rows

# Fits are available inside the $fits list
```

```
print(bootstrap_results$fits[[1]])
#> -- [ MOBSTER ] My MOBSTER model n = 400 with k = 2 Beta(s) without tail ---------------------
#>   Clusters:   = 52% [C1] and 48% [C2], with   > 0.
#>   No tail fit.
#>
#>   Beta C1 [n = 208, 52%] with mean = 0.54.
#>   Beta C2 [n = 192, 48%] with mean = 0.1.
#>   Score(s): NLL = -224.35; ICL = -409.54 (-409.54), H = 3.21 (3.21). Fit converged by MM in
#> 13 steps.
plot(bootstrap_results$fits[[1]])
#> Warning: Unknown or uninitialised column: 'Scale'.
#> Warning: Unknown or uninitialised column: 'Shape'.
#> Warning: Unknown or uninitialised column: 'Scale'.
#> Warning: Unknown or uninitialised column: 'Shape'.
#> Warning: Unknown or uninitialised column: 'Scale'.
#> Warning: Unknown or uninitialised column: 'Shape'.
#> Warning: Unknown or uninitialised column: 'Scale'.
#> Warning: Unknown or uninitialised column: 'Shape'.
#> Warning: Unknown or uninitialised column: 'Scale'.
#> Warning: Unknown or uninitialised column: 'Shape'.
#> Warning: Unknown or uninitialised column: 'Scale'.
#> Warning: Unknown or uninitialised column: 'Shape'.
```

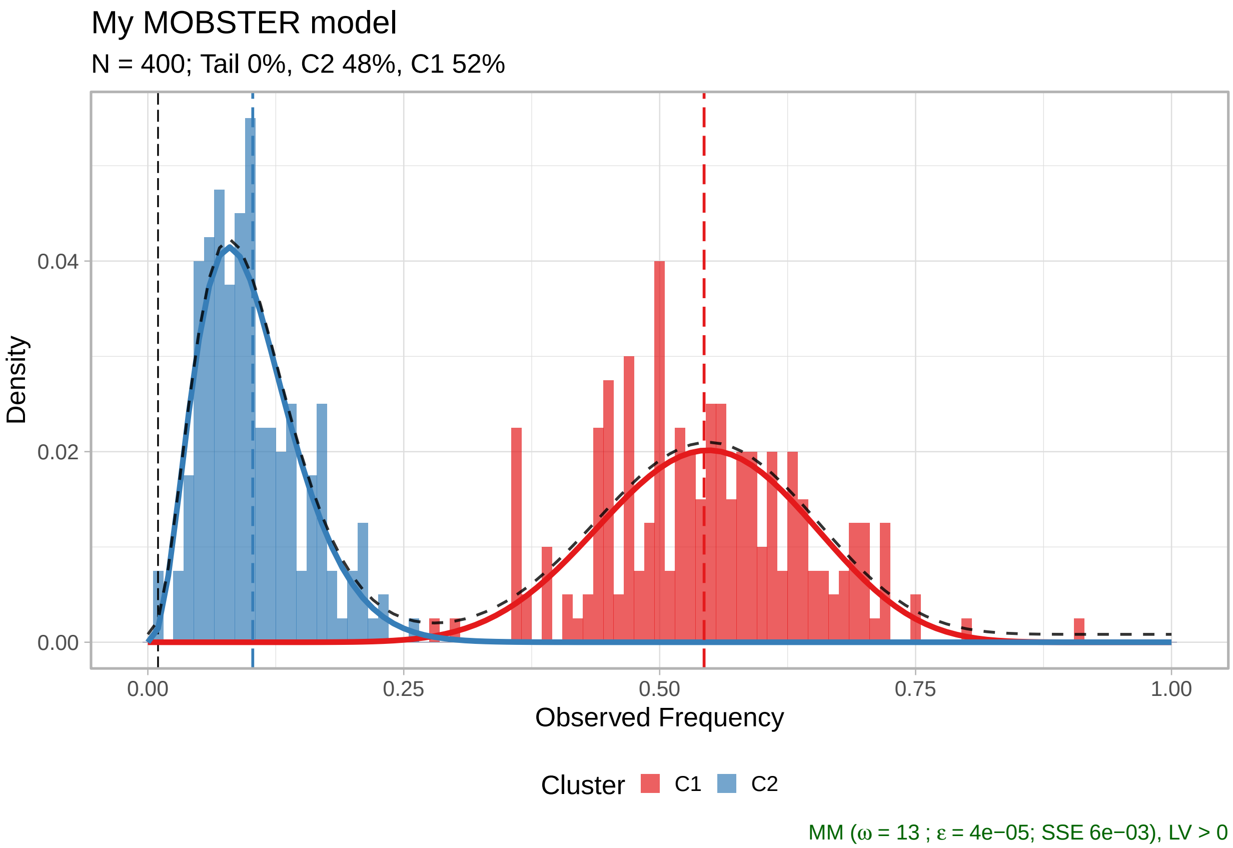

Errors of each run are available, if any.

```
print(bootstrap_results$errors)
#> NULL
```

2 Bootstrap statistics

Bootstrap statistics can be computed with `bootstrapped_statistics`.

With nonparametric bootstrap the data co-clustering probability is also computed (the probability of any pair of mutations in the data to be clustered together). Note that this probability depends on the joint resample probability of each pair of mutations (each bootstrapped with probability $1/n$, for n mutations).

`bootstrap_statistics` shows to screen several statistics.

```
bootstrap_statistics = bootstrapped_statistics(
  fit$best,
  bootstrap_results = bootstrap_results
  )
#> i Computing model frequency
#> # A tibble: 2 x 3
#>   Model               Frequency fit.model
#>   <fct>                   <dbl> <lgl>
#> 1 K = 2 without tail       0.96 TRUE
#> 2 K = 2 with tail          0.04 FALSE
#>                              v Computing model frequency ... done
#> i Confidence Intervals (CI) for empirical quantiles
#>
#> Mixing proportions
#>  # A tibble: 3 x 8
#>   cluster statistics   min lower_quantile higher_quantile   max fit.value
#>   <chr>   <chr>      <dbl>          <dbl>           <dbl> <dbl>     <dbl>
#> 1 C1      Mixing pr~ 0.465          0.472          0.541  0.548     0.506
#> 2 C2      Mixing pr~ 0.376          0.422          0.528  0.535     0.494
#> 3 Tail    Mixing pr~ 0              0              0.0579 0.145     0
#> # ... with 1 more variable: init.value <dbl>
#>
#> Tail shape/ scale
#>  # A tibble: 2 x 8
#>   cluster statistics    min lower_quantile higher_quantile    max fit.value
#>   <chr>   <chr>       <dbl>          <dbl>           <dbl>  <dbl>     <dbl>
#> 1 Tail    Scale      0.0374         0.0374          0.0374 0.0374        NA
#> 2 Tail    Shape      0.945          0.945           0.945  0.945         NA
#> # ... with 1 more variable: init.value <dbl>
#>
#> Beta peaks
#>  # A tibble: 4 x 8
#>   cluster statistics     min lower_quantile higher_quantile     max fit.value
#>   <chr>   <chr>        <dbl>          <dbl>           <dbl>   <dbl>     <dbl>
#> 1 C1      Mean       0.538          0.541           0.566   0.568     0.555
#> 2 C1      Variance   0.00803        0.00840         0.0136  0.0150    0.0109
#> 3 C2      Mean       0.0932         0.0956          0.110   0.110     0.104
#> 4 C2      Variance   0.00165        0.00189         0.00320 0.00324   0.00270
#> # ... with 1 more variable: init.value <dbl>
#>                                                v Confidence Intervals (CI) for empirical quantil
#> i Co-clustering probability from nonparametric bootstrap
```

3 Visualising bootstrap results

Object `bootstrap_statistics` contains tibbles that can be plot with specific `mobster` functions.

```
# All bootstrapped values
print(bootstrap_statistics$bootstrap_values)
#> # A tibble: 279 x 5
#>    cluster statistics        fit.value init.value resample
#>    <chr>   <chr>                 <dbl>      <dbl>    <int>
#>  1 C2      a                   3.72       0.00348        1
#>  2 C2      b                  32.6        0.00348        1
#>  3 C2      Mean                0.103      0.00599        1
#>  4 C2      Variance            0.00246    0.00376        1
#>  5 C1      a                  13.0        7.59           1
#>  6 C1      b                  10.9        7.59           1
#>  7 C1      Mean                0.543      0.188          1
#>  8 C1      Variance            0.00996    0.00368        1
#>  9 C2      Mixing proportion   0.480      0.5            1
#> 10 C1      Mixing proportion   0.520      0.5            1
#> # ... with 269 more rows

# The model probability
print(bootstrap_statistics$bootstrap_model)
#> # A tibble: 2 x 3
#>   Model              Frequency fit.model
#>   <fct>                  <dbl> <lgl>
#> 1 K = 2 without tail      0.96 TRUE
#> 2 K = 2 with tail         0.04 FALSE

# The parameter stastics
print(bootstrap_statistics$bootstrap_statistics)
#> # A tibble: 15 x 8
#>    cluster statistics       min lower_quantile higher_quantile       max
#>    <chr>   <chr>          <dbl>          <dbl>           <dbl>     <dbl>
#>  1 C1      a            8.56           9.50            15.8      16.4
#>  2 C1      b            6.96           7.74            12.6      13.5
#>  3 C1      Mean         0.538          0.541            0.566     0.568
#>  4 C1      Mixing pr~   0.465          0.472            0.541     0.548
#>  5 C1      Variance     0.00803        0.00840          0.0136    0.0150
#>  6 C2      a            2.92           2.93             4.68      5.53
#>  7 C2      b           25.0           25.4             42.3      48.8
#>  8 C2      Mean         0.0932         0.0956           0.110     0.110
#>  9 C2      Mixing pr~   0.376          0.422            0.528     0.535
#> 10 C2      Variance     0.00165        0.00189          0.00320   0.00324
#> 11 Tail    Mean       Inf            Inf              Inf       Inf
#> 12 Tail    Mixing pr~   0              0                0.0579    0.145
#> 13 Tail    Scale        0.0374         0.0374           0.0374    0.0374
#> 14 Tail    Shape        0.945          0.945            0.945     0.945
#> 15 Tail    Variance   Inf            Inf              Inf       Inf
#> # ... with 2 more variables: fit.value <dbl>, init.value <dbl>
```

Bootstrapping, one can plot the model frequency across re-samples. A model is identified by its mixture components (e.g., 2 Betas plus one tail).

```r
plot_bootstrap_model_frequency(
  bootstrap_results,
  bootstrap_statistics
  )
```

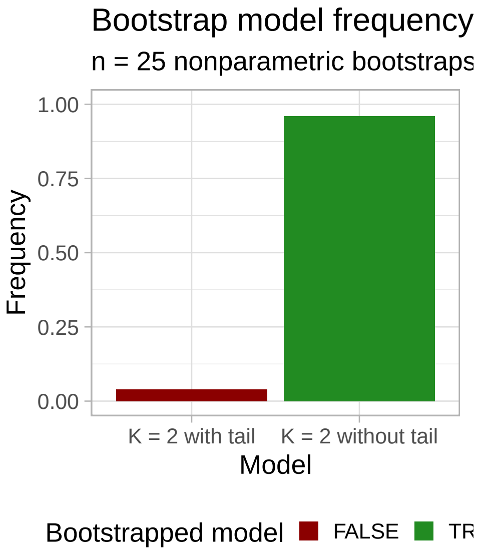

The bootstrap estimates of the parameters can be visualised.

```r
# Plot the mixing proportions
mplot = plot_bootstrap_mixing_proportions(
  fit$best,
  bootstrap_results = bootstrap_results,
  bootstrap_statistics = bootstrap_statistics
  )

# Plot the tail parameters
tplot = plot_bootstrap_tail(
  fit$best,
  bootstrap_results = bootstrap_results,
  bootstrap_statistics = bootstrap_statistics
  )

# Plot the Beta parameters
bplot = plot_bootstrap_Beta(
  fit$best,
  bootstrap_results = bootstrap_results,
  bootstrap_statistics = bootstrap_statistics
  )
#> Warning: Removed 4 rows containing missing values (geom_bar).

#> Warning: Removed 4 rows containing missing values (geom_bar).

# Figure
figure = ggpubr::ggarrange(
  mplot,
  tplot,
  bplot,
  ncol = 3, nrow = 1,
```

```r
  widths = c(.7, 1, 1)
)
#> Warning in max(data$density): no non-missing arguments to max; returning -Inf
#> Warning in max(data$density): no non-missing arguments to max; returning -Inf

print(figure)
```

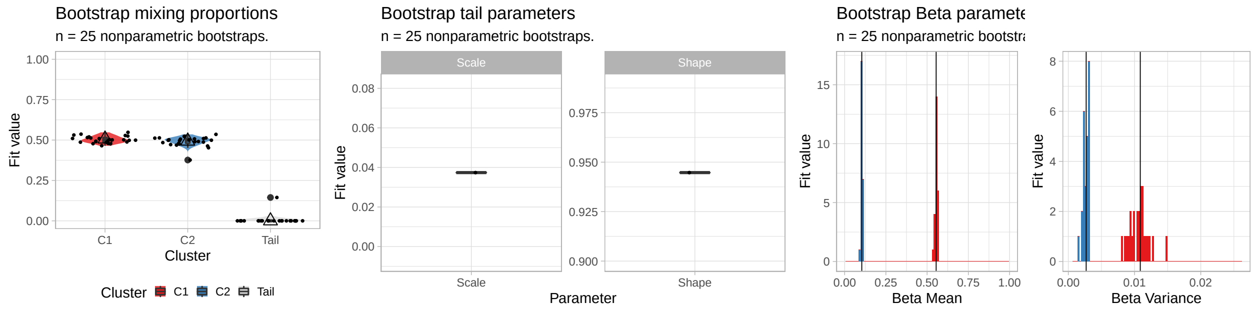

For a nonparametric bootstrap we can plot also the co-clustering probability of the data.

```r
plot_bootstrap_coclustering(
  fit$best,
  bootstrap_results = bootstrap_results,
  bootstrap_statistics = bootstrap_statistics
  )
```

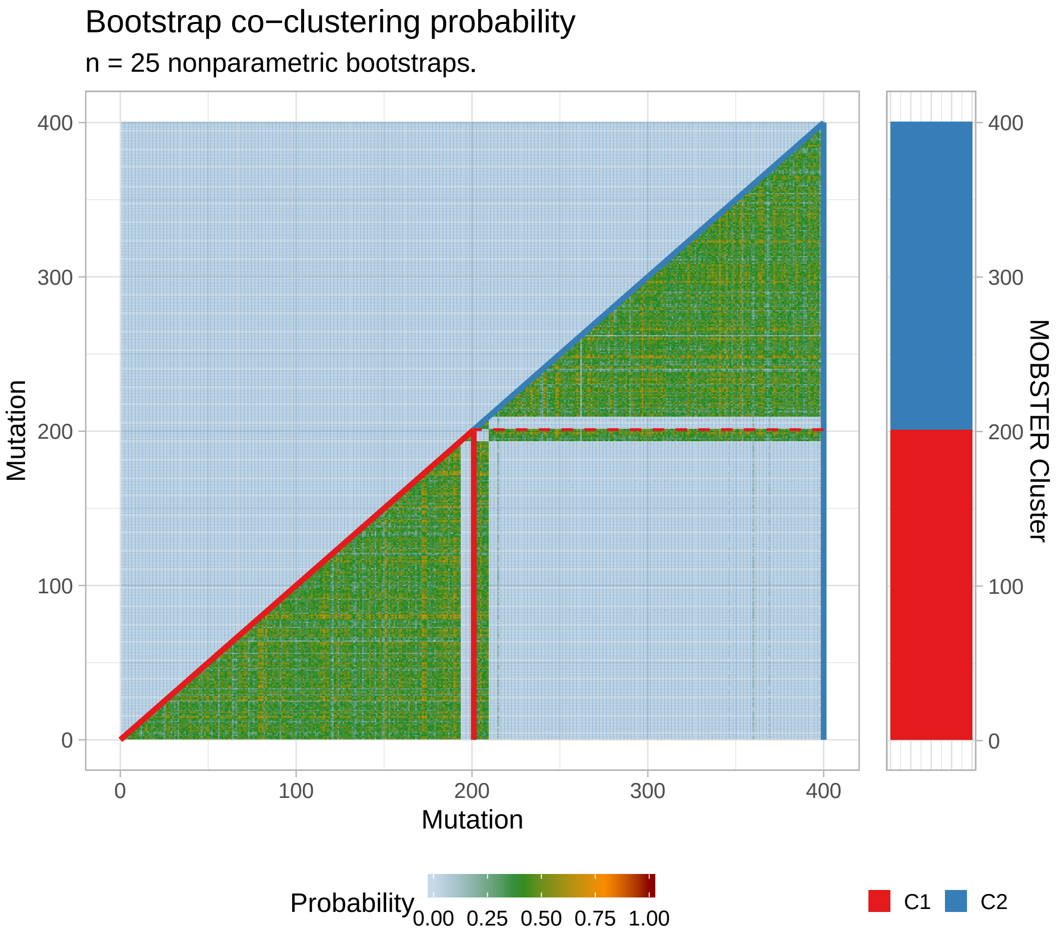

Note 4. Population Genetics statistics

```
library(mobster)
library(tidyr)
library(dplyr)
```

Population Genetics statistics can be extracted from a MOBSTER model.

```
data('fit_example', package = 'mobster')
print(fit_example$best)
#> -- [ MOBSTER ] My MOBSTER model n = 5000 with k = 2 Beta(s) and a tail ----------------------
#>   Clusters:   = 55% [C1], 31% [Tail] and 14% [C2], with   > 0.
#>   Tail [n = 1370, 31%] with alpha = 1.2.
#>   Beta C1 [n = 2784, 55%] with mean = 0.48.
#>   Beta C2 [n = 846, 14%] with mean = 0.15.
#>   Score(s): NLL = -5671.5; ICL = -10359.09 (-11266.35), H = 907.26 (0). Fit converged by MM
#> in 75 steps.

evolutionary_parameters(fit_example)
#> # A tibble: 1 x 7
#>      mu exponent  time subclonefrequency subclonemutations cluster     s
#>   <dbl>    <dbl> <dbl>             <dbl>             <dbl> <chr>   <dbl>
#> 1  73.5     2.25  5.98             0.298              695. C2      0.177
```

The mutation rate `mu` (cell division units) scaled by the probability of lineage survival β, μ/β, is given by:

$$\mu/\beta = \frac{M}{(\frac{1}{f_{\min}} - \frac{1}{f_{\max}})}$$

Where $f_{\min}$ is the minimum VAF and $f_{\max}$ is the maximum, and M is the number of mutations between $f_{\min}$ and $f_{\max}$.

Selection is defined as the relative growth rates of host tumour cell populations (λh) vs subclone (λs):

$$1 + s = \frac{\lambda h}{\lambda s}$$

The mathematical details of these computations are described in the main paper, and baesd on the population genetics model of tumour evolutionin Williams et al. 2016 and 2018 (Nature Genetics).

5. Clone-specific dN/dS statistics

```
library(mobster)
library(tidyr)
library(dplyr)
```

1 Computing dnds values

`mobster` interfaces with the dndscv R package to compute dN/dS values from its output clusters. The method implemented in `dndscv` is described in *Martincorena, et al. "Universal patterns of selection in cancer and somatic tissues"*, Cell 171.5 (2017): 1029-1041; PMID 29056346).

Requirements. In order to be able to compute dN/dS values mutations data must store their *genomic coordinates*:

- chromosome location `chrom`,
- position `from`,
- reference alleles `alt` and `ref`.

Besides, it is important to know what is the reference genome used to align the genome; this information will be used by `dndscv` to annotate input mutations.

We show this analysis with the fits for one of the lung samples available in the package.

```
fit = mobster::LUFF76_lung_sample

# Print and plot the model
print(fit$best)
#> -- [ MOBSTER ]  n = 2298 with k = 1 Beta(s) and a tail ---------------------------------------
#>   Clusters:   = 50% [Tail] and 50% [C1], with   > 0.
#>   Tail [n = 1076, 50%] with alpha = 2.
#>   Beta C1 [n = 1222, 50%] with mean = 0.25.
#>   Score(s): NLL = -3095.24; ICL = -5784.22 (-6144.04), H = 359.82 (0). Fit converged by MM
#> in 51 steps.
plot(fit$best)
```

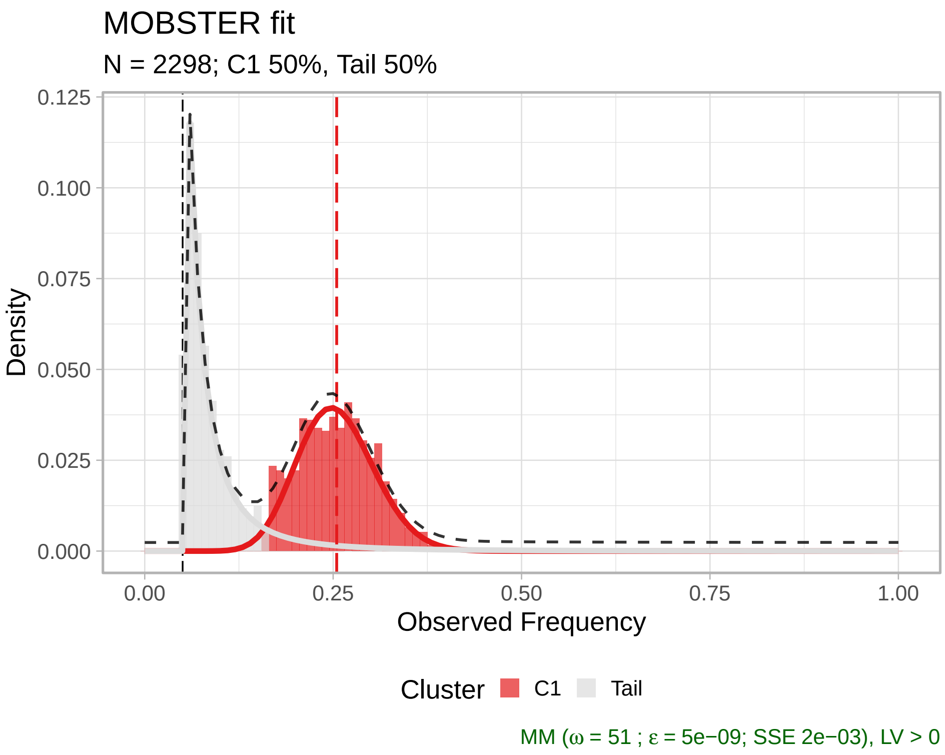

We compute the values using the clustering assignments from the best fit.

```
clusters = Clusters(fit$best)
print(clusters)
#> # A tibble: 2,298 x 14
#>      Key Callers t_alt_count t_ref_count Variant_Classif~    DP    VAF chr
#>    <int> <chr>         <int>       <int> <chr>            <int>  <dbl> <chr>
#>  1   330 mutect~          23         149 intergenic         172 0.134  chr2
#>  2   331 mutect~          53         133 intergenic         186 0.285  chr2
#>  3   332 mutect~           8         132 intergenic         140 0.0571 chr2
#>  4   334 mutect~          26         112 intergenic         138 0.188  chr2
#>  5   335 mutect~          40         102 intronic           142 0.282  chr2
#>  6   336 mutect~          32          95 intronic           127 0.252  chr2
#>  7   337 mutect~          28         134 intergenic         162 0.173  chr2
#>  8   338 mutect~          14         148 intergenic         162 0.0864 chr2
#>  9   339 mutect~          11         108 intronic           119 0.0924 chr2
#> 10   340 mutect~          11         171 intergenic         182 0.0604 chr2
#> # ... with 2,288 more rows, and 6 more variables: from <chr>, ref <chr>,
#> #   alt <chr>, cluster <chr>, Tail <dbl>, C1 <dbl>
```

The available clusters are `C1` and `Tail`; `C1` is the clonal cluster. We compute dN/dS with the default parameters.

```
# Run by cluster and default gene list
dnds_stats = dnds(
  clusters,
  gene_list = NULL
)
#> Missing 'sample' column, assuming mutations from a single patient (adding a sample label otherwise).
#> i 2298 mutations; 'by cluster' groups in 0 samples, with no genes (default dndscv).
#> [refdb = hg19] Removing chr from chromosome names for hg19 reference compatability
```

```
#>
#>   C1 Tail
#> 1222 1076
#>
#> -- Running dndscv ------------------------------------------------------------------------------
#>
#> -- Group Tail
#> [1] Loading the environment...
#> [2] Annotating the mutations...
#> [3] Estimating global rates...
#> [4] Running dNdSloc...
#> [5] Running dNdScv...
#>     Regression model for substitutions (theta = 6.69e-05).
#>
#> -- Group C1
#> [1] Loading the environment...
#> [2] Annotating the mutations...
#> [3] Estimating global rates...
#> [4] Running dNdSloc...
#> [5] Running dNdScv...
#> dndscv error
#> Error in while ((it <- it + 1) < limit && abs(del) > eps) {: missing value where TRUE/FALSE needed
#> -- dndscv results ------------------------------------------- wall, wmis, wnon, wspl, wtru --
#> # A tibble: 5 x 5
#>   name            mle cilow cihigh dnds_group
#>   <chr>         <dbl> <dbl>  <dbl> <chr>
#> 1 wmis  0.731         0.113   4.73 Tail
#> 2 wnon  0.00000000804 0      Inf    Tail
#> 3 wspl  0.0000000260  0      Inf    Tail
#> 4 wtru  0.0000000170  0      Inf    Tail
#> 5 wall  0.701         0.109   4.51 Tail
```

The statistics can be computed for a custom grouping of the clusters. Here it does not make much difference because we have only the clonal cluster, and the tail; but if we had one subclone `C2` we could have pooled together the mutations in the clones using

```
# Not run here
dnds_stats = dnds(
  clusters,
  mapping = c(`C1` = 'Non-tail', `C2` = 'Non-tail', `Tail` = 'Tail'),
  gene_list = NULL
)
```

In the above analysis we have run `dndscv` using the default gene list (`gene_list = NULL`). Notice that errors raised by `dndscv` are intercepted by `mobster`; some of this errors might originate from a dataset with not enough substitutions to compute dN/dS.

The call returns:

- the table computed by `dndscv`, where column `dnds_group` labels the group.
- a `ggplot` plot of the point estimates and the confidence interval;

```
# Summary statistics
print(dnds_stats$dnds_summary)
#> # A tibble: 5 x 5
#>   name            mle cilow cihigh dnds_group
```

```
#>    <chr>          <dbl> <dbl>  <dbl> <chr>
#> 1 wmis  0.731          0.113   4.73 Tail
#> 2 wnon  0.00000000804 0      Inf     Tail
#> 3 wspl  0.0000000260  0      Inf     Tail
#> 4 wtru  0.0000000170  0      Inf     Tail
#> 5 wall  0.701          0.109   4.51 Tail

# Table observation countns
print(dnds_stats$dndscv_table)
#> # A tibble: 20,091 x 15
#>    gene_name n_syn n_mis n_non n_spl wmis_cv wnon_cv wspl_cv pmis_cv ptrunc_cv
#>    <chr>     <dbl> <dbl> <dbl> <dbl>   <dbl>   <dbl>   <dbl>   <dbl>     <dbl>
#>  1 ATF4          0     1     0     0   5262.       0       0 1.00e-4     0.998
#>  2 C14orf79      0     1     0     0   4702.       0       0 1.13e-4     0.998
#>  3 FAM189A1      0     1     0     0   3155.       0       0 1.72e-4     0.997
#>  4 RNF150        0     1     0     0   2793.       0       0 1.96e-4     0.998
#>  5 NEO1          0     1     0     0   1141.       0       0 5.09e-4     0.994
#>  6 TTN           0     0     0     0      0        0       0 8.36e-1     0.968
#>  7 MUC16         0     0     0     0      0        0       0 8.95e-1     0.990
#>  8 OBSCN         0     0     0     0      0        0       0 9.15e-1     0.989
#>  9 SYNE1         0     0     0     0      0        0       0 9.17e-1     0.985
#> 10 NEB           0     0     0     0      0        0       0 9.19e-1     0.978
#> # ... with 20,081 more rows, and 5 more variables: pallsubs_cv <dbl>,
#> #   qmis_cv <dbl>, qtrunc_cv <dbl>, qallsubs_cv <dbl>, dnds_group <chr>

# Plot
print(dnds_stats$plot)
```

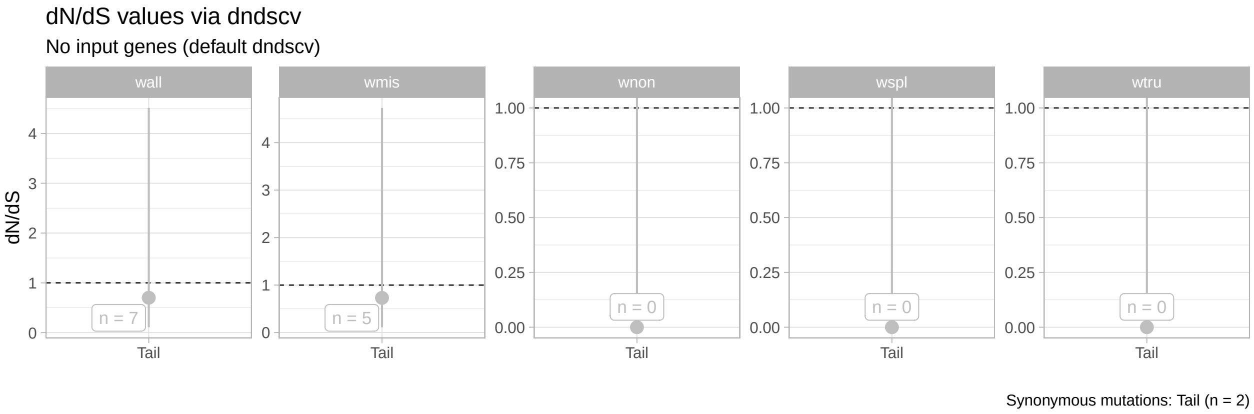

The default plot contains results obtained from all substitution models available in `dndscv`. Specific models can be required using the parameters of the `dnds` function.

2 Using custom genes lists

A custom list of genes can be supplied in the call to `dnds` as the variable `genes_list`; the package provides 4 lists of interests for this type of computation:

- a list of driver genes compiled in `Martincorena et al. Cell 171.5 (2017): 1029-1041.`;
- a list of driver genes compiled in `Tarabichi, et al. Nature Genetics 50.12 (2018): 1630.`;
- a list of essential genes compiled in `Wang et al. Science 350.6264 (2015): 1096-1101.`;

- a list of essential genes compiled in `Bloomen et al. Science 350.6264 (2015): 1092-1096.`.

which are available to load.

```
# Load the list
data('cancer_genes_dnds', package = 'mobster')

# Each sublist is a list
print(lapply(cancer_genes_dnds, head))
#> $Martincorena_drivers
#> [1] "CCDC6"     "EIF1AX"    "HIST1H2BD" "MED12"     "POLE"      "SMARCB1"
#>
#> $Tarabichi_drivers
#> [1] "ACVR1"  "ACVR1B" "AKT1"   "ALK"    "AMER1"  "APC"
#>
#> $Wang_essentials
#> [1] "ABL1"    "RPL23A"  "AARS2"   "TRMT112" "FARSA"   "ABCB7"
#>
#> $Bloomen_essentials
#> [1] "AARS"     "AASDHPPT" "AATF"     "ABCB7"    "ABCE1"    "ABCF1"
```

A custom gene list can be used as follows.

```
# Not run here
dnds_stats = dnds(
  clusters,
  mapping = c(`C1` = 'Non-tail', `C2` = 'Non-tail', `C3` = 'Non-tail', `Tail` = 'Tail'),
  gene_list = cancer_genes_dnds$Martincorena_drivers
)
```

3 Pooling data from multiple patients

The input format of the `dnds` function allows to pool data from several fits at once. We pool data from the 2 datasets available in the package.

```
# 2 lung samples
data('LU4_lung_sample', package = 'mobster')
data('LUFF76_lung_sample', package = 'mobster')
```

We pool the data selecting the required columns.

```
dnds_multi = dnds(
  rbind(
    Clusters(LU4_lung_sample$best) %>% select(chr, from, ref, alt, cluster) %>% mutate(sample = 'LU4'),
    Clusters(LUFF76_lung_sample$best) %>% select(chr, from, ref, alt, cluster) %>% mutate(sample = 'LUFF
  ),
  mapping = c(`C1` = 'Non-tail',  # Pool together all clonal mutations
              `Tail` = 'Tail'     # Pool together all tail mutations),
  )
)
#> i 3580 mutations; 2 groups in 2 samples, with no genes (default dndscv).
#> [refdb = hg19] Removing chr from chromosome names for hg19 reference compatability
#>
#> Non-tail     Tail
#>     2194     1386
```

```
#>
#> -- Running dndscv ------------------------------------------------------------------------------
#>
#> -- Group Non-tail
#> [1] Loading the environment...
#> [2] Annotating the mutations...
#> [3] Estimating global rates...
#> [4] Running dNdSloc...
#> [5] Running dNdScv...
#>     Regression model for substitutions (theta = 17.5).
#>
#> -- Group Tail
#> [1] Loading the environment...
#> [2] Annotating the mutations...
#> [3] Estimating global rates...
#> [4] Running dNdSloc...
#> [5] Running dNdScv...
#>     Regression model for substitutions (theta = 8.54).
#> -- dndscv results ------------------------------------------ wall, wmis, wnon, wspl, wtru --
#> # A tibble: 10 x 5
#>    name             mle  cilow cihigh dnds_group
#>    <chr>          <dbl>  <dbl>  <dbl> <chr>
#>  1 wmis  1.16          0.375    3.58 Non-tail
#>  2 wnon  2.25          0.223   22.8  Non-tail
#>  3 wspl  0.0000000301  0       Inf   Non-tail
#>  4 wtru  0.658         0.0716   6.05 Non-tail
#>  5 wall  1.11          0.370    3.33 Non-tail
#>  6 wmis  0.508         0.121    2.13 Tail
#>  7 wnon  0.00000000835 0       Inf   Tail
#>  8 wspl  0.0000000461  0       Inf   Tail
#>  9 wtru  0.0000000201  0       Inf   Tail
#> 10 wall  0.472         0.115    1.95 Tail

# Plot
print(dnds_multi$plot)
```

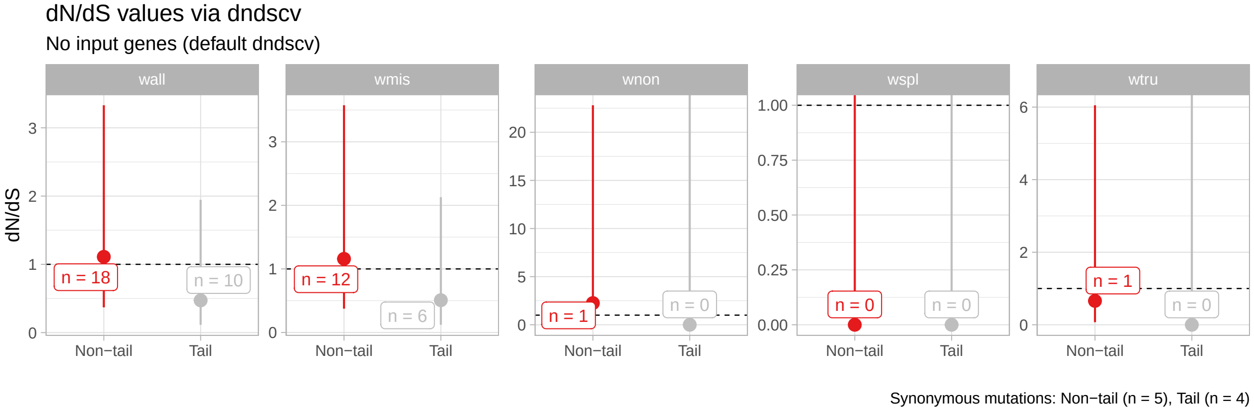

6. Computing clone trees from clusters

```
library(mobster)
library(tidyr)
library(dplyr)
```

Clone trees from `mobster` fits can be computing using the internal interface with ctree.

You need to have drivers annotated your object if you want to use `ctree`, and every `driver_label` has to be unique, as it will be used as the `variantID` column to identify the driver event.

We show the analysis with a synthetic dataset.

```
# Example data where we annotate 3 events as drivers
example_data = Clusters(mobster::fit_example$best)

# Drivers annotation
drivers_rows = c(2239, 3246, 3800)

example_data$is_driver = FALSE
example_data$driver_label = NA

example_data$is_driver[drivers_rows] = TRUE
example_data$driver_label[drivers_rows] = c("DR1", "DR2", "DR3")

# Fit and print the data
fit = mobster_fit(example_data, auto_setup = 'FAST')
#>  [ MOBSTER fit ]
#>  Loaded input data, n = 5000.
#>  n = 5000. Mixture with k = 1,2 Beta(s). Pareto tail: TRUE and FALSE. Output clusters with
#>  > 0.02 and n > 10.
#> ! mobster automatic setup FAST for the analysis.
#>  Scoring (without parallel) 2 x 2 x 2 = 8 models by reICL.
#>  MOBSTER fits completed in 7.4s.
#> -- [ MOBSTER ] My MOBSTER model n = 5000 with k = 2 Beta(s) and a tail ----------------------
#>  Clusters:   = 55% [C1], 31% [Tail] and 14% [C2], with   > 0.
#>  Tail [n = 1370, 31%] with alpha = 1.2.
#>  Beta C1 [n = 2784, 55%] with mean = 0.48.
#>  Beta C2 [n = 846, 14%] with mean = 0.15.
#>  Score(s): NLL = -5671.5; ICL = -10359.09 (-11266.35), H = 907.26 (0). Fit converged by MM
#> in 75 steps.
#>  The fit object model contains also drivers annotated.
#> # A tibble: 3 x 7
#>      VAF cluster   Tail       C1       C2 is_driver driver_label
#>    <dbl> <chr>    <dbl>    <dbl>    <dbl> <lgl>     <chr>
#> 1 0.448  C1      0.0125 9.88e- 1 8.08e-21 TRUE      DR1
#> 2 0.159  C2      0.225  2.35e-34 7.75e- 1 TRUE      DR2
#> 3 0.0629 Tail    1.00   1.91e-82 4.02e- 5 TRUE      DR3

best_fit = fit$best
```

```r
print(best_fit)
#> -- [ MOBSTER ] My MOBSTER model n = 5000 with k = 2 Beta(s) and a tail ----------------------
#>   Clusters:   = 55% [C1], 31% [Tail] and 14% [C2], with   > 0.
#>   Tail [n = 1370, 31%] with alpha = 1.2.
#>   Beta C1 [n = 2784, 55%] with mean = 0.48.
#>   Beta C2 [n = 846, 14%] with mean = 0.15.
#>   Score(s): NLL = -5671.5; ICL = -10359.09 (-11266.35), H = 907.26 (0). Fit converged by MM
#> in 75 steps.
#>   The fit object model contains also drivers annotated.
#> # A tibble: 3 x 7
#>      VAF cluster   Tail       C1       C2 is_driver driver_label
#>    <dbl> <chr>    <dbl>    <dbl>    <dbl> <lgl>     <chr>
#> 1 0.448  C1      0.0125 9.88e- 1 8.08e-21 TRUE      DR1
#> 2 0.159  C2      0.225  2.35e-34 7.75e- 1 TRUE      DR2
#> 3 0.0629 Tail    1.00   1.91e-82 4.02e- 5 TRUE      DR3
```

1 Tree computation

Tree computation removes any mutation that is assigned to a `Tail` cluster because the clone tree represents the clones.

```r
# Get the trees, select top-rank
trees = get_clone_trees(best_fit)
#> v Loading ctree, 'Clone trees in cancer'. Support : <https://caravagn.github.io/ctree/>
#>  [ ctree ~ generate clone trees for My_MOBSTER_model ]
#> Sampler :  10000 (cutoff),  5000 (sampling),  100 (max store)
#> # A tibble: 2 x 5
#>   cluster    R1 nMuts is.clonal is.driver
#>   <chr>   <dbl> <dbl> <lgl>     <lgl>
#> 1 C2      0.149   846 FALSE     TRUE
#> 2 C1      0.478  2784 TRUE      TRUE
#>
#>   Region R1  ~  #CCF clusters > 1%:  2
#>   Trees per region 1
#>   Hashed trees 1
#> There are no alternatives!
#>   Pigeonhole Principle
#>   Ranking trees
#>   Trees with non-zero sscore 1 storing 1
```

The *top-rank* tree is in position `1` of `trees`; `ctree` implements S3 object methods to print an plot a tree.

```r
top_rank = trees[[1]]

# Print with S3 methods from ctree
ctree:::print.ctree(top_rank)
#>  [ ctree - ctree rank 1/1 for My_MOBSTER_model ]
#>
#> # A tibble: 2 x 5
#>   cluster    R1 nMuts is.clonal is.driver
#>   <chr>   <dbl> <dbl> <lgl>     <lgl>
#> 1 C2      0.149   846 FALSE     TRUE
#> 2 C1      0.478  2784 TRUE      TRUE
```

```
#>
#> Tree shape (drivers annotated)
#>
#>   \-GL
#>    \-C1 :: DR1
#>     \-C2 :: DR2
#>
#> Information transfer
#>
#>    DR1 ---> DR2
#>    GL ---> DR1
#>
#> Tree score 1
```

We can plot the top tree, aggregating different `ctree` plots.

```
# 1) Clone tree
# 2) Input ctree data (here adjusted VAF)
# 3) Clone size barplot
ggpubr::ggarrange(
  ctree::plot.ctree(top_rank),
  ctree::plot_CCF_clusters(top_rank),
  ctree::plot_clone_size(top_rank),
  nrow = 1,
  ncol = 3
)
```

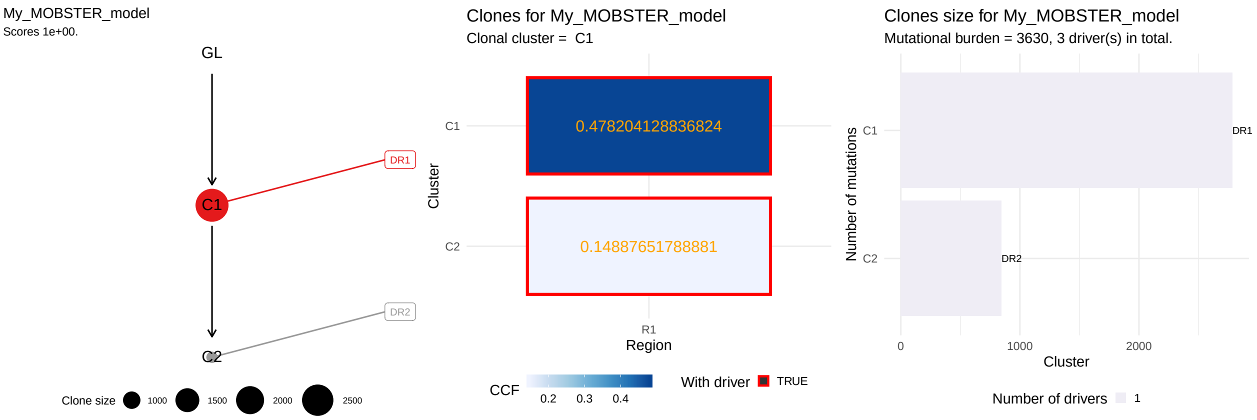

Supplement: Supplementary file 1 — Additional file 1. Supplementary notes that describe the software and its applications. [file 12859_2020_3863_MOESM1_ESM.pdf]
